# Inter- and intra-species variation in genome-wide gene expression of *Drosophila* in response to parasitoid wasp attack

**DOI:** 10.1186/s12864-017-3697-3

**Published:** 2017-04-27

**Authors:** Laura Salazar-Jaramillo, Kirsten M. Jalvingh, Ammerins de Haan, Ken Kraaijeveld, Henk Buermans, Bregje Wertheim

**Affiliations:** 1 Groningen Institute for Evolutionary Life Sciences, University of Groningen, Nijenborgh 7, Groningen, 9700 CC The Netherlands; 2 Institute of Evolutionary Biology, The University of Edinburgh, Charlotte Auerbach Road, Edinburgh, EH9 3FL UK; 3 Department of Ecology and Evolution, University of Lausanne, Biophore, Lausanne, CH-1015 Switzerland; 4 Centre for Neural Circuits and Behaviour, University of Oxford, Mansfield Road, Oxford, OX1 3SR UK; 5 Animal Ecology, Department of Ecological Sciences, VU University Amsterdam, De Boelelaan 1085, Amsterdam, 1081 HV The Netherlands; 6 Leiden Genome Technology Center, Department of Human Genetics, Leiden University Medical Center, Einthovenweg 20, Leiden, 2333 ZC The Netherlands

**Keywords:** RNAseq, Drosophila speciesm, Evolution immune response, Parasitoid wasp

## Abstract

**Background:**

Parasitoid resistance in *Drosophila* varies considerably, among and within species. An immune response, lamellocyte-mediated encapsulation, evolved in a subclade of *Drosophila* and was subsequently lost in at least one species within this subclade. While the mechanisms of resistance are fairly well documented in *D. melanogaster*, much less is known for closely related species. Here, we studied the inter- and intra-species variation in gene expression after parasitoid attack in *Drosophila*. We used RNA-seq after parasitization of four closely related *Drosophila* species of the melanogaster subgroup and replicated lines of *D. melanogaster* experimentally selected for increased resistance to gain insights into short- and long-term evolutionary changes.

**Results:**

We found a core set of genes that are consistently up-regulated after parasitoid attack in the species and lines tested, regardless of their level of resistance. Another set of genes showed no up-regulation or expression in *D. sechellia*, the species unable to raise an immune response against parasitoids. This set consists largely of genes that are lineage-restricted to the melanogaster subgroup. Artificially selected lines did not show significant differences in gene expression with respect to non-selected lines in their responses to parasitoid attack, but several genes showed differential exon usage.

**Conclusions:**

We showed substantial similarities, but also notable differences, in the transcriptional responses to parasitoid attack among four closely related *Drosophila* species. In contrast, within *D. melanogaster*, the responses were remarkably similar. We confirmed that in the short-term, selection does not act on a pre-activation of the immune response. Instead it may target alternative mechanisms such as differential exon usage. In the long-term, we found support for the hypothesis that the ability to immunologically resist parasitoid attack is contingent on new genes that are restricted to the melanogaster subgroup.

**Electronic supplementary material:**

The online version of this article (doi:10.1186/s12864-017-3697-3) contains supplementary material, which is available to authorized users.

## Background

Comparative biology has contributed enormously to the understanding of evolution [1]. In the last decade the comparative approach has extended to genomes, enabling us to study how phenotypic diversity is encoded in the genome. Although the relationship between genotype and phenotype is complex, current Next Generation Sequencing technology has made a great contribution in genotype-phenotype mapping. It allows for the inspection of whole genomes and transcriptomes in a relatively unbiased way, and it enables investigations beyond model species. We can now extend the comparison of traits that have been long studied in one model species to closely related species, and to lines experimentally selected for changes in traits, in order to better understand the evolution of that trait [2]. In this way we can contrast differences between species and lines of the same species to gain insights into long and short term evolutionary changes.

One trait that shows remarkably fast evolution and dramatic changes among species is the immune response to parasites. The hosts’ ability to defend against parasites has to continuously evolve and re-adjust to the co-evolving parasites [3]. These hosts’ defense mechanisms often consist of specific immune responses that effectuate the clearance of the parasite. When the hosts co-evolve with their local parasite communities, they may change the investment, or even acquire novel immunity traits in the arms’ race with the parasites [4].

In insects, immune responses against parasitoid wasps have evolved multiple times [5]. Parasitoids are insects that lay eggs on or in other arthropods, killing the host during the development. They are among the most abundant and species-rich arthropod groups, and due to their lethality, exert a strong selection pressure on their hosts [6]. Insect species are often natural hosts to different parasitoid species [7], showing large variation in the mechanisms and effectiveness of their immune responses, even among closely related species and among natural populations [5, 8–11].

The mechanism of immune response against parasitoids has been well documented in *D. melanogaster*. It involves the recognition of the parasitoid egg by the host, followed by an increase and differentiation of hemocytes (blood cells in invertebrates) that surround the egg, leading to the formation of multicellular capsule, which is melanized. The differentiation of hemocytes is a critical step in the process, and produces three main types of cells: 1) lamellocytes, which are responsible for forming the capsule around the egg, and are usually not present in unparasitized larvae 2) plasmatocytes, which phagocytize small pathogens and can differentiate further into lamellocytes [12] and 3) crystal cells, which contain the melanin that is deposited in the capsule [13]. The immune response against parasitoid is associated with variation in the constitutive or induced numbers of hemocytes per species within the melanogaster group in *Drosophila*. Generally, higher constitutive hemocyte loads or stronger induction correlate to higher levels of immunity [8]. A similar pattern was found after experimental selection for increased parasitoid resistance within a single population of *D. melanogaster* [14, 15]. Interestingly, however, natural populations of *D. melanogaster* that vary in parasitoid resistance did not show this correlation, possibly suggesting that the genetic basis for the fine-tuning of the immune responses has evolved differently among various populations [16].

The changes in the gene expression that mediate this immune response have been investigated with microarrays in *D. melanogaster* [14, 17, 18]. An important conclusion from these studies was that lines that were experimentally selected for increased resistance to parasitoids showed changed expression in a large number of genes, but did not evolve a constitutive increase in expression of immune genes. This implies that the evolved increased immunity in the selection lines did not consist of a simple pre-activation of the inducible immune response, but involved a different set of genes (i.e., the set of genes differentially expressed upon parasitization differed largely from the set of genes differentially expressed constitutively in the selected lines) [14]. Moreover, comparisons of the genome sequences after the experimental selection for increased parasitoid resistance and control lines revealed significant changes in large numbers of genome regions, suggesting that at short evolutionary timescales, the modulation of immune responses against parasitoids acts mostly on standing genetic variation across a large number of loci [19].

For *Drosophila* species other than *D. melanogaster*, less information is available about the mechanisms and genes involved in the immune response against parasitoids. In a previous study we showed that this type of immune response and the lamellocytes that mediate the encapsulation, are restricted to a monophyletic clade within the melanogaster group and has been secondarily lost in one species within this group, *D. sechellia* [20]. By comparing the sequences of genes differentially expressed during the immune response after parasitoid attack in *D. melanogaster* across multiple species, we hypothesized that this novel trait involved the recruitment of new genes on existing immune pathways [20]. To infer whether these genes are indeed associated with the evolution of this trait in the melanogaster lineage, we first need to verify that they are also associated with the immune response after parasitoid attack in the other species. By characterizing the transcriptional responses to parasitoid attack in *Drosophila* species, we can contrast how short- and long-term evolution affect expression of (the same or different) genes.

In this study we aim to test the hypothesis that short- and long-term evolutionary processes may lead to substantial dissimilarities in the genetic basis of a trait. We use a comparative approach to characterize how (dis)similar the transcriptional response is to an immune challenge, i.e. parasitoid attack, in four closely related species and in four different strains of a single species. These *Drosophila* species and lines all rely on lamellocyte-mediated encapsulation for the immune response against parasitoid, but differ substantially in their level of immunity against parasitoids. We compared the transcriptional activity during the immune response after parasitoid attack, using RNAseq, in different species (*D. melanogaster, D. simulans, D. sechellia* and *D. yakuba*) and in experimentally selected lines of *D. melanogaster*. We analysed gene expression in *Drosophila* larvae after parasitoid attack and their respective controls at two different time points, 5 and 50 hours after parasitization, which reflect the start and end of the immune response [17]. The specific questions we addressed are 1) which genes change in expression in response to attack by the parasitoid *Asobara tabida* in these four closely related species? 2) what are the similarities and differences in the transcriptional responses compared to *D. melanogaster* 3) how short-term selection processes for higher parasitoid resistance affect gene expression during the immune response after parasitoid attack? and 4) how does the function and orthology of differentially expressed genes reflect the evolutionary history of the immune response against parasitoids?

## Results

**Fig. 1:**
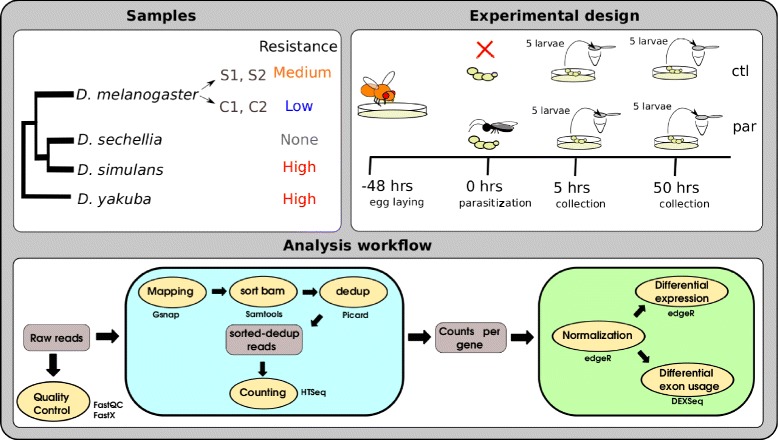
Experimental design and workflow. Schematic representation of samples, experimental design and analysis workflow

The RNA-seq experiment consisted of 84 samples from four species, *D. melanogaster*, *D. simulans*, *D. sechellia* and *D. yakuba*. For *D. melanogaster* four lines were used, two selected for increased parasitoid resistance and two non-selected. For each of the other three species one line was used. Gene expression in larvae was compared between parasitized and non-parasitized treatments at two time points, 5 and 50 hours (5h and 50h) after parasitization. These time points correspond to the peaks of gene expression in the initiation and completion of the immune response after parasitoid attack in *D. melanogaster* [17]. Three biological replicates were obtained for each line, treatment and time point. These lines and species differed in their level of resistance against parasitoids (Fig. 1).

**Fig. 2:**
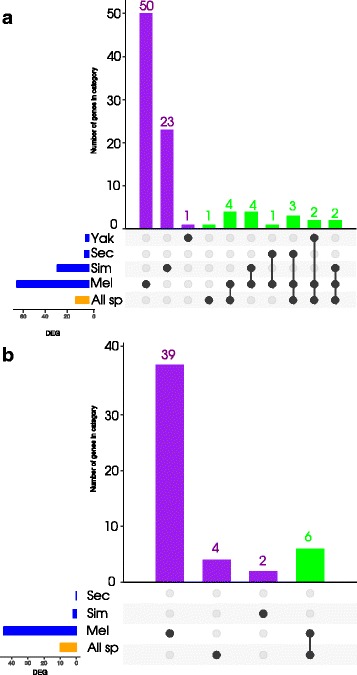
Summary of the number of significant DEG. Summary of the number of significant DEG in each contrast **a** for 5h and **b** for 50h after parasitoid attack. The number of DEG is shown in horizontal barplots (blue: species-specific normalization, yellow: all-species normalization). Vertical barplots show the overlap in differentially expressed genes in the categories indicated by the filled dots

Sequenced reads were processed as shown in Fig. 1 to obtain estimates of gene expression for each gene. Normalization and differential gene expression analysis on the counts was analysed using the package edgeR (version 3.12.0) [21] implemented in R (version 3.2.4). In order to study the intra- and inter-species variation, we implemented two alternative normalizations: 1) a species-specific normalization to investigate the response of each species independently, using four lines of *D. melanogaster* or one line for the remaining species and 2) an all-species normalization to investigate the common response among all species, using one line per species and the orthologous genes to *D. melanogaster*. Differential gene expression was considered significant at a False Discovery Rate (FDR) threshold <0.05 (Additional file 1: Figure S5, Additional file 2: Figure S6, Additional file 3: Figure S7 and Additional file 4: Figure S8). A summary of the number of differentially expressed genes (DEG) between parasitized and non-parasitized larvae in the different analyses is shown in Fig. 2.

### Gene expression in response to parasitoid attack in four *Drosophila* species

Unfortunately, the power to detect differences in gene expression was very low in the single-species analyses, except for *D. melanogaster*. For the latter we could combine four lines (two selection and two control), which with replicates resulted in 12 parasitized samples (and 12 respective controls) per time point, while we only had three parasitized samples (and three respective controls) per time point for the other species.

When comparing the gene expression in response to parasitoid attack for each species separately, we found great differences in the number of DEG in *D. melanogaster* compared to the other species. At 5h and 50h, respectively, the number of DEG was 66 and 45 in *D. melanogaster* (Additional file 5: Figure S9 and Additional file 6: Figure S10), 31 and 2 in *D. simulans* (Additional file 7: Figure S11 and Additional file 8: Figure S12), 4 and 0 in *D. sechellia* (Additional file 7: Figure S11) and 3 and 0 in *D. yakuba* (Additional file 7: Figure S11). This is largely explained by the difference in samples sizes (lines) per species (see above).

### Between-species variation

To search for similarities and differences in genome-wide responses to parasitoid attack among the 4 *Drosophila* species, we had to increase the power of comparison across species without introducing bias due to differences in the number of lines. We normalized all species together (genes were annotated to their ortholog in *D. melanogaster*) and used only one line of *D. melanogaster*, “C1” (the other lines of *D. melanogaster* were also tested, and the resulting DEG was a subset of C1). The statistical analysis was applied to 8394 genes that were present in all species. We found large variation in overall patterns of gene expression between species, with gene expression in *D. yakuba* samples being least similar to the other species (Additional file 9: Figure S1, Additional file 10: Figure S2, Additional file 11: Figure S3 and Additional file 12: Figure S4). The expression distance between *D. yakuba* and all other species at 50h appeared to have been enhanced by technical problems during sequencing of one batch, and consequently this species was discarded for further analysis at the 50h time point.

**Fig. 3:**
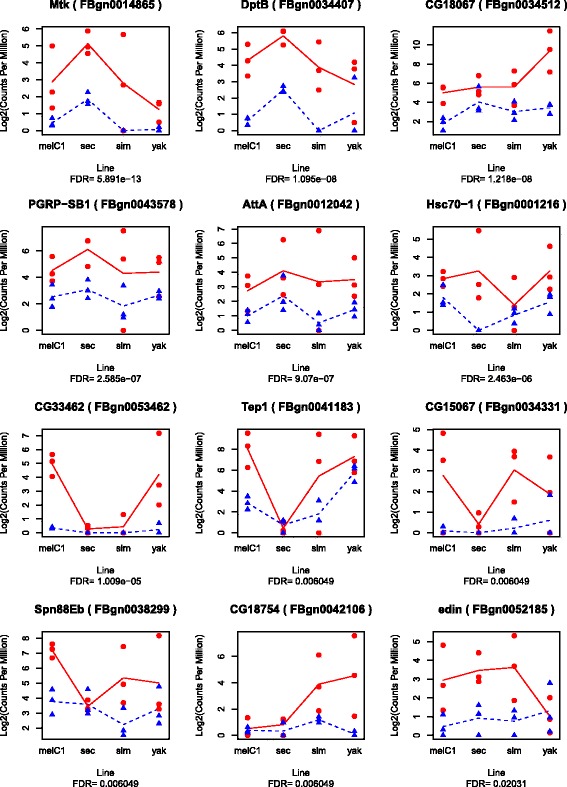
Expression of the significantly DEG after parasitoid attack in 4 *Drosophila* species at 5h. (Log2-transformed) counts per million (CPM) of parasitized (*red dots*) and control (*blue triangles*)

The analysis of the data that was normalized with all species led to 12 and 7 DEG at 5h and 50h, respectively (Fig. 3 and. 4). Some of these genes were consistently up-regulated in all species whereas other genes were up-regulated only in a subset of the species, suggesting that the addition or exclusion of species influenced the statistical significance of DEG. To investigate this, we analysed subsets of species (*D. melanogaster*-*D. simulans* and *D. melanogaster*-*D. simulans*-*D. sechellia*), and found 26 and 9 significant DEG at 5h and 50h, respectively, including the significant DEG found for all species (labeled as “spClade" for a subset and “spAll” for genes found in all species in Additional file 13: Table S1). The number of DEG increased with the increase of species, particularly in the subset *D. melanogaster*-*D. simulans*; but decreased when including *D. yakuba* (at 5h; this species was removed at 50h). This may be a consequence of the similarity in the response shared among *D. melanogaster* and *D. simulans*, and the greater dissimilarity in the response of *D. yakuba*.

**Fig. 4:**
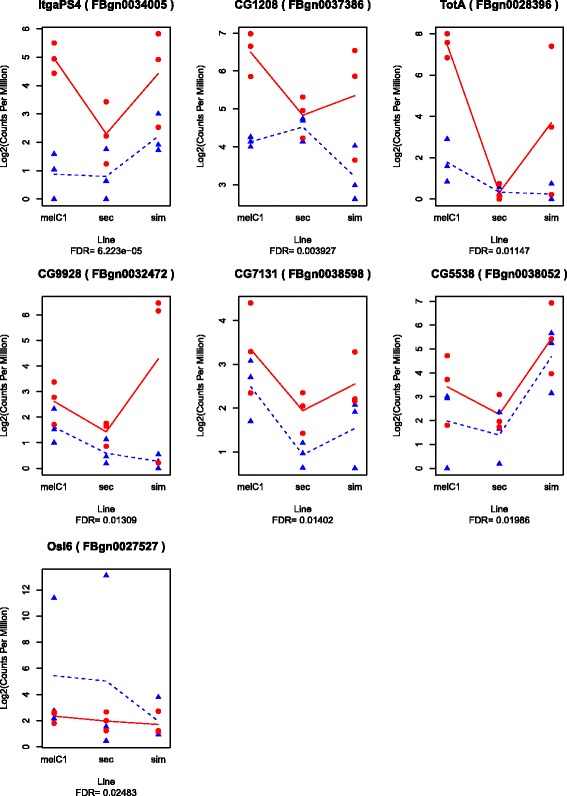
Expression of the significantly DEG after parasitoid attack in 4 *Drosophila* species at 50h. (Log2-transformed) counts per million (CPM) of parasitized (*red dots*) and control (*blue triangles*)

Contrasting the two normalizations thus led to different subsets of DEG. Some of the significant DEG found in the per-species analyses overlapped with the all-species normalization at 5h. The increase in the number of DEG by combining species confirms that the low numbers of DEG in the per-species analysis is indeed largely due to difference in statistical power. It also indicates that the genetic basis of the immune response to parasitoid attack is at least partially shared among the four species. At 50h, the number of differentially expressed genes was smaller for the all-species normalization and no overlap was found with the per-species normalization (Fig. 2). No statistical power was gained by combining different species, indicating that the completion of the immune response was more variable among the species.

### Within-species variation after selection for increased parasitoid resistance

**Fig. 5:**
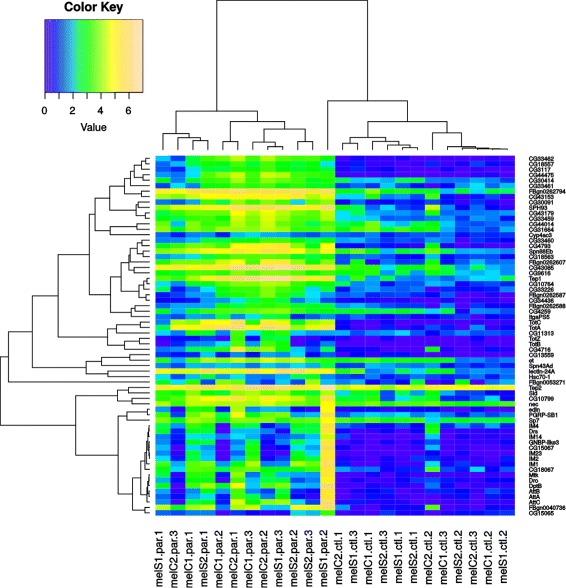
Heatmap of significantly DEG in *D. melanogaster* at 5h. Colors represent the CPM values for all lines, treatments and replicates. The first 12 columns present the gene expression in larvae that were parasitized (par) in lines that had been selected (S) for increased resistance and their respective unselected control lines (C), and the second 12 columns the expression in not-parasitized control (ctl) larvae

**Fig. 6:**
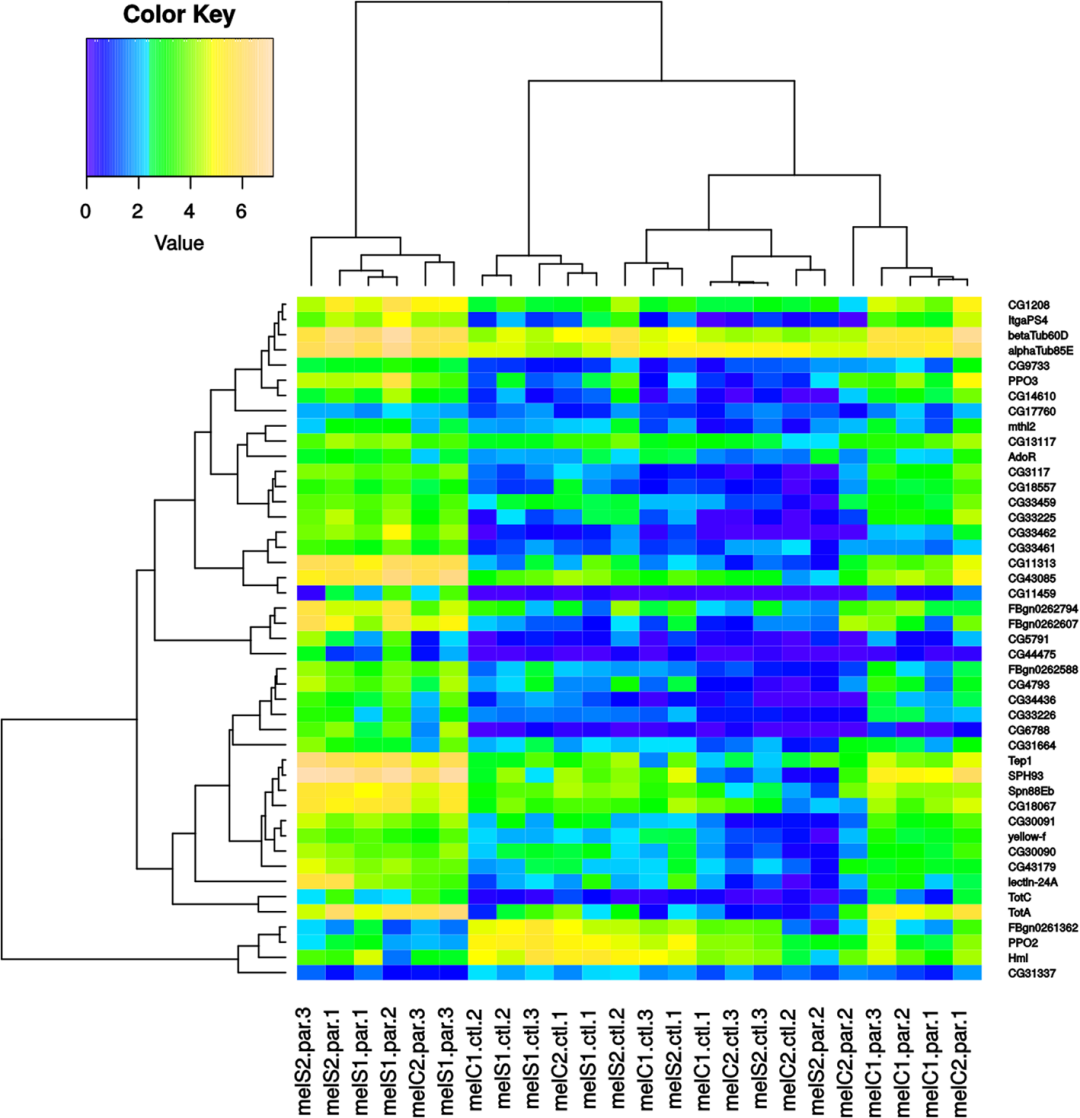
Heatmap of significantly DEG in *D. melanogaster* at 50h. Colors represent the CPM values for all lines, treatments and replicates. The first 12 columns present the gene expression in larvae that were parasitized (par) in lines that had been selected (S) for increased resistance and their respective unselected control lines (C), and the second 12 columns the expression in not-parasitized control (ctl) larvae

When focusing on the gene expression within *D. melanogaster*, we found that all lines initiated the response against the parasitoid attack in a similar way (Fig. 5), but that the termination of the response was less consistent across lines (Fig. 6). The clustering did not reveal a clear pattern in terms of resistance (e.g. non-selected vs selected lines) among the significant DEG due to parasitization. Thus, to better understand the effect of selection, we tested two models. One that considered constitutive differences in expression (before parasitization) and another that considered the interaction between selection and parasitization. These two models were tested for each time point independently. Neither of the models showed significantly differential expression between selection and control lines (Additional file 5: Figure S9 and Additional file 6: Figure S10).

For *D. melanogaster* we also analysed the differential exon usage, which reflects the expression of transcript isoforms. Exon usage was compared using the R package DEXSeq [22] between 1) parasitized and non-parasitized (“par”) samples, 2) selected and non-selected (“sel”) samples and 3) the interaction between parasitized and selected (“int”). Genes showing significant differential exon usage are listed in Table 1 (see Additional file 14: Figure S13, Additional file 15: Figure S14, Additional file 16: Figure S15, Additional file 17: Figure S16, Additional file 18: Figure S17, Additional file 19: Figure S18, Additional file 20: Figure S19, Additional file 21: Figure S20 and Additional file 22: Figure S21 for the plots of exon coverage). Three of these genes, *CG15065, Tep2* and *CG4133* were also found to be differentially expressed after parasitization in *D. melanogaster*.

**Table 1:** List of genes with significant differential exon usage in *D. melanogaster*

Significant genes	Contrast
FBgn0040734 (CG15065)	par
FBgn0041182 (Tep2)	par,int
FBgn0262607 (CG43133)	par,int
FBgn0263773 (fok)	sel
FBgn0014469 (CG2060)	sel
FBgn0038247 (CG3389)	int
FBgn0067629+FBgn0067628 (CG33332)	int

### Orthologous and functional annotations of differentially expressed genes

**Fig. 7:**
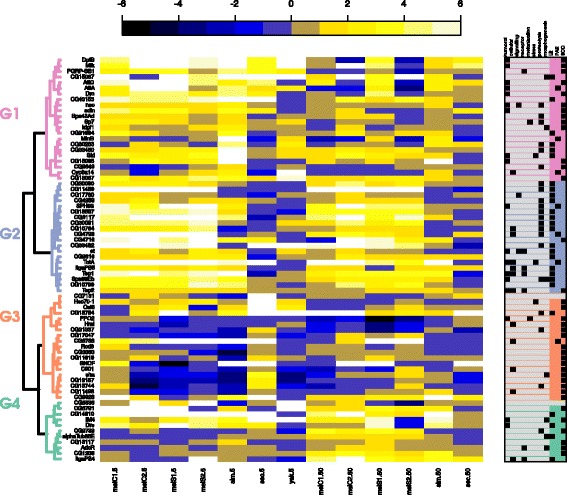
Heatmap of DEG from all analyses, species and time points. Genes were included only if they were differentially expressed in at least one species, line or timepoint, and were expressed in all species (49 genes were excluded that were not present or not expressed in some species). The data for *D. yakuba* at 50h is excluded due to technical problems. The colors of the heatmap indicate the (log2-transformed) median fold change of the three replicates. The fold change was calculated as the ratio of counts per million of parasitized to control samples. The distance matrix was calculated using Pearson correlation and clustered using “ward.D2”. Each gene was annotated with eight functional and three orthologous categories (*right panel*, where the presence is indicated by a *black filled squares*)

Together all models and normalizations produced 121 DEG (Additional file 23: Table S2), of which 72 genes were found expressed in all species, lines and time points (the remaining 49 were not present or not expressed in some species). We classified these 72 genes in eight broad functional categories, according to their Gene Ontology (Flybase, GeneOntology): “humoral”, “cellular”, “signalling”, “receptors”, “melanization”, “stress”, “proteolysis” and “morphogenesis” (Additional file 13: Table S1); and in three general orthologous classes according to their presence-absence pattern across *Drosophila* species (taken from OrthoDB, which is based on the 12 species with whole sequenced genomes): “single copy ortholog” (SCO) for genes that have exactly one copy in each species, “paralog” (PAR) for genes with multiple orthologs in more than two species, and “lineage restricted” (LR) for those genes present only in a (monophyletic) subset of species. These genes were clustered based on the median (log2-transformed) fold changes in expression of parasitized versus control samples (Fig. 7).

Cluster G1 is composed largely of humoral, single-copy-orthologous genes, that showed strong up-regulation at 5h for most species, regardless of the level of resistance. Some genes did not show up-regulation in *D. sechellia*, the species that has lost the immune response against parasitoid. Other genes were not up-regulated in *D. yakuba*. At 50h, most genes did no longer show strong up-regulation, except for some that remained strongly up-regulated in *D. simulans*. The genes in this cluster may be considered a core set of immune genes that react to a general immune challenge, including parasitoids.

Cluster G2 is composed of genes with strong up-regulation at both 5h and 50h in *D. melanogaster* and *D. simulans*. This set contains proteolytic and immune genes of both the humoral and cellular response, which are mostly lineage-restricted. In *D. sechellia*, almost none of these genes were differentially expressed, and also in *D. yakuba* only a few of the genes were up-regulated at 5h after parasitoid attack. Thus, this set includes mostly genes that are restricted to the clade that can launch an immune response against parasitoids, and some of the genes are up-regulated in all 3 species with this immune response, but not in *D. sechellia*.

Cluster G3 is composed of down-regulated genes at both 5h and 50h mostly in *D. melanogaster*, and consists largely of single-copy-orthologous genes, some in the cellular immune response. Finally, cluster G4 contains a subset of genes that show upregulation mostly at 50h in *D. melanogaster* and *D. simulans*, but not in *D. sechellia*. These genes consist of both single-copy-orthologous and lineage-restricted genes, with various functional annotations.

## Discussion

In order to contrast how short- and long-term evolution affect gene expression, we compared intra- and inter-species differences in genome-wide gene expression after parasitoid attack using RNA-seq. We studied four *Drosophila* species: *D. simulans*, *D. sechellia*, *D. yakuba* and *D. melanogaster*, for the latter species we compared two lines selected for higher resistance to parasitoids with two non-selected lines. These species and lines differ in their ability to immunologically respond to the infection by parasitoid wasps. We found that phenotypic differences between species were to a large extent reflected in the expression profiles. The species that had lost parasitoid resistance, *D. sechellia*, failed to upregulate several genes that were upregulated by the resistant species. Interestingly, also the three resistant species showed substantial variation in transcriptional responses to parasitoid attack: some genes were upregulated by only a subset of these species, or exclusively in one of the species. In contrast, within *D. melanogaster*, the responses of the selection and control lines was remarkably similar, despite their phenotypic differences in resistance.

### Intraspecific variation in gene expression

This study follows up on previous work on *D. melanogaster* using microarrays and re-sequencing [14, 17, 19]. Besides extending the knowledge beyond the model species *D. melanogaster*, the comparison of results with different techniques (e.g., microarrays vs RNAseq) is a valuable practice as it informs us about the robustness of conclusions previously drawn. The microarray studies implicated several hundreds of genes that changed expression during the response to parasitoid attack [17, 18], and during larval development in selection lines with increased resistance (without exposure to parasitoids) [14]. The genome sequencing revealed sequence changes that occurred during the evolution of increased parasitoid resistance [19]. In the current study, we investigated how gene expression in these selection lines responded after parasitization. We identified 96 genes that were differentially expressed in response to parasitoid attack in *D. melanogaster* and 121 in total across the 4 species. Of all these genes, 52 had also been identified by the microarray experiment that compared parasitized vs non-parasitized larvae, 14 with the microarray experiment that compared selected vs non-selected lines and 10 with the genome sequencing comparing selected vs non-selected lines (Additional file 23: Table S2). The overlap between the results of microarrays and RNA-seq approaches is considerable, indicating robustness of previous findings on the changes in gene expression in response to parasitoid attack.

In the current study, we did not find genes that were significant differentially expressed when comparing selected vs non-selected *D. melanogaster* lines. The absence of significant genes between selected vs non-selected lines was surprising since 900 genes with constitutive differential expression were previously found [14]. There are several possible explanations for this discrepancy. First, the studies measured different aspects: the (constitutive) changes during development of the larvae that were less susceptible but without exposing them to parasitoid attack (microarrays) versus the (induced) changes in response to parasitoid attack (current study). Secondly, the studies had different designs: we used three biological replicates per line for 2 time points, while the earlier microarray study used five biological replicates for 7 time points. Thus, we may have had insufficient power to detect subtle differences between lines that would have been detected in the microarray study. Thirdly, the microarray study used only one selected line, and some of the significant genes in that study may therefore have been caused by random genetic drift rather than by selection. Finally, different biases are inherent to microarray and RNA-seq. One difficulty of RNA-seq is to understand whether anomalies in coverage are biological or are due to technical artifacts arising from library preparation [23]. Although normalization methods should account for this, it has been shown that the variation introduced by the different steps can cause strong over- or under-estimation of gene expression [24], which hinders the power to detect differential expression, in particular when such differences are small. On the other hand, microarrays have a low sensitivity for detecting differential expression in genes that are expressed at low levels, low power for genes that are constitutively highly expressed, and are limited to the genes that are represented on the array.

In contrast to the absence of significant differences in expression between selected vs non-selected, we found a high level of replication between parasitized vs non-parasitized in the current study and the microarray study by [17]. This may be the consequence of a highly canalized response, producing more consistent differences in gene expression across lines and different species, especially at early stages of the immune response. Additionally, the fold-changes in gene expression in response to parasitoid attack are generally larger, which perhaps suggests that the analysis of RNA-seq data is more robust for the detection of large fold change differences in expression than more subtle changes.

In our RNA-seq study we investigated differences in transcript structure, which was not possible in previous microarrays. We showed that seven genes had differential exon usage, three of which were also differentially expressed. Although it is not yet clear how the use of transcript isoforms affects the efficiency of the response, this could expose an additional molecular level where selection can potentially act. Changes in exon-usage may affect protein-protein interactions in a molecular network, while the phenotypic outcome depends largely on epistatic interactions among genes within the existing genetic networks. In order to understand short-term selection and the extent to which selection experiments are repeatable at a molecular level, it is important to consider a wide range of levels, from exons, to genes, gene families, pathways and protein-protein interaction networks.

### Interspecific variation in gene expression

The current study also revealed that many of the genes that were differentially expressed during the immune response against parasitoids in *D. melanogaster* showed similar transcriptional responses in closely related *Drosophila* species. At the inter-species level we found that the expression profiles of *D. melanogaster* and *D. simulans* were most similar to each other, as expected given their phylogenetic proximity. The large dissimilarities in the transcriptional responses of *D. sechellia*, which lost this immune response, also supports the importance of these genes in the immune response against parasitoid.

The current study also revealed a number of genes that were differentially expressed in *D. melanogaster* in response to parasitoid attack, but not in both of the two other species that have strong immune responses. These genes could either be less closely associated to the immune function, or they could indicate dissimilarity in the genetic basis of immunity among species. It was, for example, intriguing to find substantial dissimilarity in the expression profile of *D. yakuba* in response to parasitoid attack. It thus appears that *D. yakuba* regulates its immune response differently from *D. melanogaster* and *D. simulans*, although the three species show a phenotypically similar immune response to parasitoids [20]. Some genes that were upregulated by *D. melanogaster* and *D. simulans* did not change expression in *D. yakuba* (e.g. *edin*), while others were already highly expressed in unparasitized *D. yakuba* (e.g., *Tep1*). It is relevant to note that *D. yakuba* had the highest and fastest resistance during the phenotypic characterization ([20], personal observation), which could mean that part of the patterns may be explained by the timing of the transcriptional response (e.g. that *D. yakuba* had already completed its immune response). Interestingly, some species of the obscura and ananassae group are able to resist parasitoids through a very different mechanism, using different types of hemocytes [25, 26], which is likely to have evolved independently [20]. Future gene expression studies on these more distant groups could be valuable to understand the molecular basis of these independently evolved responses to parasitoid attack.

In this study we were able to characterize general patterns that may be common in the immune response against parasitoids. When combining all species and all lines together (Fig. 7), we found a core set of immune genes that was activated early in the response, regardless of the host resistance level. Most of these genes are humoral and are conserved across the *Drosophila* genus [20]. A second set of genes was activated throughout the response, except in *D. sechellia* (and to a lesser extent in *D. yakuba*). Interestingly, this set contains six of the genes we had previously identified as new and fast changing in the *Drosophila* clade, that can resist parasitoids through melanotic encapsulation (*CG30090, CG4259, CG4793, TotA, Tep1, Spn88EB* [20]). These genes may reflect an immune response that is more specific to parasitoid attack, and are therefore key for further studies. Finally, there is a number of genes that are differentially expressed after parasitization in *D. melanogaster* and *D. simulans*, but not in *D. yakuba*. Perhaps these genes are not required for inducing the immune response itself, but have been recruited during the co-evolution with parasitoids by the ancestor of *D. simulans* and *D. melanogaster*, possibly modulating the immune response or mitigating costs of the immune response.

Essential to the immune response against parasitoids is the proliferation, differentiation and migration of hemocytes that form the capsule around the parasitoid egg. Similarly to previous studies [17, 18] we found no differential expression of genes involved in the hemopoiesis process. Since hemopoiesis is an important component of the development, it is possible that changes that lead to differential expression are selected against during evolution. The levels and differentiation of hemocyte could be regulated by other molecules with less pleiotropic effects. For example, we did find genes involved in melanization and cell adhesion, both of which are important for the cellular response. Several of these genes were down-regulated (e.g., *Hml, PPO2, CG6788, alphaTub85E*) or were only activated in the later time point (e.g., *ItgaPS4, PPO3*). In particular, we were able to confirm the lack of expression of the lamellocyte specific prophenoloxidase *PPO3* in *D. sechellia* (no counts were obtained for reads mapping to the corresponding region in the genome). We previously proposed that *PPO3* is non-functional in *D. sechellia*, which correlated with the lack of immunological and transcriptional response in this species [20]. Recently, loss of function of the *PPO3* gene was indeed shown due to an inactivating mutation which introduces a stop codon, generating a truncated version of the protein [27].

## Conclusion

In conclusion, this study provides an important dataset to understand the evolution of the immune response against parasitoids in *Drosophila*. We showed both notable similarities and differences among closely related *Drosophila* species in their transcriptional responses to parasitoid attack. Based on this we can now generate various hypotheses that need to be tested with additional experimental approaches. While replicating previous studies in *D. melanogaster* in a combined experiment, we also report for the first time the gene expression profiles after parasitoid attack in *D. simulans*, *D. sechellia* and *D. yakuba*. Our results show that in the short-term, selection does not seem to act on a pre-activation of the immune response (as previously suggested in [14]). Instead, it may target alternative pathways or mechanisms (e.g., exon usage), which have an impact on the coordination and speed of the response. Across *Drosophila* species, the immune response against parasitoid involves different sets of genes: 1) a core of immune genes that expresses early and independent of the level of resistance of the species, and are mostly conserved and 2) a set of immune and proteolytic genes, which are mostly lineage-restricted to the melanogaster subgroup while 3) not all species that can resist parasitoids in this melanogaster subgroup show the same changes in expression for the same set of genes. This supports the hypothesis the encapsulation ability in the melanogaster subgroup depends on the expression of new genes that act on existing pathways.

## Methods

### Insect strains

For the comparison within a species, two lines of *D. melanogaster* selected for increased resistance and two control lines were used. These lines and the artificial selection procedure are described in [19]. Briefly, the common base population for these lines was originally collected in Leiden, The Netherlands and had a low level of resistance. For each selection line the second-instar larvae were exposed to a moderately virulent strain of the parasitoid *A. tabida* for 24 hours. After pupation each individual was manually checked under a stereo-microscope and only those pupae that contained a visible capsule (sign of parasitization and resistance) and survived to adulthood were taken to the next generation. Selection was applied for five generations. Alongside each selection line, a matched control line (“non-selected line”) was cultured in parallel. Lines differed significantly in their resistance, with selected lines showing an average resistance of 50% and non-selected lines 20% at the end of the experimental selection procedure. The sampling for the RNA-seq experiment was performed 31 generations after the discontinuation of the experimental selection procedure, while the selection lines were still significantly more resistant than the control lines.

For the comparison across species, genome project strains were used for *D. simulans*, *D. yakuba* and *D. sechellia* from the *Drosophila* Stock Center (San Diego University) [28]. The immune response of these strains against parasitoids is described in [20].

For the parasitizations, the *A. tabida* parasitoid strain, “TMS”, was used. This inbred line was established as an isofemale line in 2010 from a cross between two lines, one originally from Sospel (France) and one from Pisa (Italy). It has a moderate level of virulence.

Flies were reared at 20° under a dark:light regime of 12:12 and 50% relative humidity in quarter-pint bottles containing 30 mL standard medium (26 g dried yeast, 54 g sugar, 17 g agar and 13 mL nipagine solution per litre). The parasitoid *A. tabida* has been maintained on *D. melanogaster* at 20° under a dark:light regime of 12:12.

### Parasitization

Fifty second-instar *Drosophila* larvae of each species were exposed to one *A. tabida* female. Larvae for which parasitoid oviposition was observed for at least 10 seconds were transferred to a new petri dish to allow development for a fixed period of time (5 hours or 50 hours) after which sampling took place. A control group was treated in the same way, except no wasp was introduced. Three biological replicates, each consisting of five larvae, were taken per species or line, treatment and time point. This resulted in a total of 84 samples for RNA-seq analysis.

### RNA extraction

Larvae were collected, immediately snap frozen in liquid nitrogen and stored at −80°. The RNA was extracted for pools of five larvae, using ZR Tissue & Insect RNA MicroPrep ^*TM*^ kit (Zymo Research), according to the manufacturers’ instructions. The RNA was eluted in RNase free water. Quality control was performed with Nanodrop of (260/280 and 260/230 close or above 2) and Bioanalyzer. Samples having a Bioanalyzer concentration of preferably 2*μ* *g*, but at least 1*μ* *g* in 15*μ* *l* and showing strong, distinct peaks corresponding to 18S and 28S rRNA were accepted for sequencing.

### Sequencing

Sequencing was performed in June-August 2012 in the Leiden Genome Technology Center. Three pooled libraries were constructed, containing one biological replicate for each species, line, treatment (parasitized or control) and time point (5h or 50h after parasitization). Each sample in the libraries was individually barcoded (barcodes were randomly assigned). Strand specific RNA-seq libraries were generated using the method described by [29] with minor modifications. In short, mRNA was isolated from 500 ng total RNA using oligo-dT Dynabeads (LifeTech 61002) and fragmented to 150–200 nt in first strand buffer for 3 minutes at 94°. Random hexamer primed first strand was generated in presence of dATP, dGTP, dCTP and cTTP. Second strand was generated using dUTP instead of dTTP to tag the second strand. Subsequent steps to generate the sequencing libraries were performed with the NebNext kit for Illumina sequencing with minor modifications, i.e., after indexed adapter ligation to the dsDNA fragments, the library was treated with USER enzyme (NEB M5505L) in order to digest the second strand derived fragments. After amplification of the libraries, samples with unique sample indexes were pooled and paired-end 2x100 bp sequenced on 1 single HiSeq2000-v3 lane. Each pooled library was sequenced on 2 lanes, and a 7th lane was used to re-run some of the failed samples.

### Analysis

Quality control of the raw reads was performed with the Fastx toolkit (http://hannonlab.cshl.edu/fastx_toolkit/). Reads were trimmed based on a Phred Score smaller than 20 using FastQC 0.013 (http://www.bioinformatics.babraham.ac.uk/projects/fastqc/). Filtered reads were mapped using Gsnap (parameters: -B 3 -t 6 -A sam) [30] to the respective reference genome downloaded from FlyBase (dmel5.51, dsim2.01, dsec1.3 and dyak1.3). The alignments were run in the Millipede Cluster of Groningen University and the BlaxterLab cluster in Edinburgh University. Sam and bam files were manipulated with Samtools [31] and duplicated reads were removed with Picard Tools 1.79 (http://broadinstitute.github.io/picard/). Counts were calculated with HTSeq-counts [32] based on uniquely mapped and unambiguous reads only. Differential expression of the counts was analysed using the Bioconductor package edgeR (version 3.12.1) [21]. We tested two glm functions implemented in the edgeR package glmFit (glmLRT), which uses a chi-square approximation to the likelihood ratio statistic and glmQLFit (glmQLFTest), which uses a quasi-likelihood F-test. Although the second approach makes fewer assumptions, it was too conservative for our biological variation and experimental design. We therefore present here the results from the glmLRT and include in the supplementary material (Additional file 13: Table S1) the results using the glmQLT function. Differential exon usage was analysed using the Bioconductor package DEXSeq (1.14.2) [22]. Annotation files were modified to match the annotation file of *D. melanogaster*, using the orthology file from Flybase (*gene*_*orthologs*_*fb*_2013_06.*tsv*).

## Additional files

Additional file 1 MA Plot for all species 5h. Log-fold change against log-counts per million for all species at 5h. (PDF 338 kb)

Additional file 2 MA Plot for all species 50h. Log-fold change against log-counts per million for all species at 50h. (PDF 337 kb)

Additional file 3 MA Plot for *D. melanogaster* at 5h. Log-fold change against log-counts per million for *D. melanogaster* at 5h. (PDF 436 kb)

Additional file 4 MA Plot for *D. melanogaster* at 50h. Log-fold change against log-counts per million for *D. melanogaster* at 50h. (PDF 432 kb)

Additional file 5 CPM Plot for *D. melanogaster* at 5h. Log2 counts per million of control (blue triangles) and parasitized (red circles) for *D. melanogaster* at 5h. (PDF 39 kb)

Additional file 6 CPM Plot for *D. melanogaster* at 50h. Log2 counts per million of control (blue triangles) and parasitized (red circles) for *D. melanogaster* at 50h. (PDF 28 kb)

Additional file 7 CPM Plot for *D. simulans, D. sechellia, D. yakuba* at 5h. Log2 counts per million of control (blue triangles) and parasitized (red circles) for *D. simulans, D. sechellia, D. yakuba* at 5h. (PDF 9 kb)

Additional file 8 CPM Plot for *D. simulans* at 50h. Log2 counts per million of control (blue triangles) and parasitized (red circles) for *D. simulans* at 5h. (PDF 5 kb)

Additional file 9 MDS Plot for all species 5h. Multidimensional scaling plot showing gene expression distances for all species at 5h. (PDF 5 kb)

Additional file 10 MDS Plot for all species 50h. Multidimensional scaling plot showing gene expression distances for all species at 50h. (PDF 5 kb)

Additional file 11 MDS Plot for *D. melanogaster* 5h. Multidimensional scaling plot showing gene expression distances for *D. melanogaster* 5h. (PDF 5 kb)

Additional file 12 MDS Plot for *D. melanogaster* 50h. Multidimensional scaling plot showing gene expression distances for *D. melanogaster* 50h. (PDF 5 kb)

Additional file 13 Functional categories. Classification in meta-categories based on Gene Onthology annotation. (DOCX 11 kb)

Additional file 14 Differential exon usage of CG15065 “par”. Fitted expression value of CG15065 showing in pink the exon with significant differential expression. (PDF 18 kb)

Additional file 15 Differential exon usage Tep2 “par”. Fitted expression value of Tep2 showing in pink the exon with significant differential expression. (PDF 37 kb)

Additional file 16 Differential exon usage Tep2 “int”. Fitted expression value of Tep2 showing in pink the exon with significant differential expression. (PDF 39 kb)

Additional file 17 Differential exon usage CG43133 “par”. Fitted expression value of CG43133 showing in pink the exon with significant differential expression. (PDF 22 kb)

Additional file 18 Differential exon usage CG43133 “int”. Fitted expression value of CG43133 showing in pink the exon with significant differential expression. (PDF 23 kb)

Additional file 19 Differential exon usage of fok “sel”. Fitted expression value of fok showing in pink the exon with significant differential expression. (PDF 28 kb)

Additional file 20 Differential exon usage CG2060 “sel”. Fitted expression value of CG2060 showing in pink the exon with significant differential expression. (PDF 30 kb)

Additional file 21 Differential exon usage CG3389 “int”. Fitted expression value of CG3389 showing in pink the exon with significant differential expression. (PDF 28 kb)

Additional file 22 Differential exon usage CG33332 “int”. Fitted expression value of CG33332 showing in pink the exon with significant differential expression. (PDF 40 kb)

Additional file 23 List of all significant differentially expressed genes. FBgn: Flybase symbol; Symbol _mel: symbol of orthologous gene in *D. melanogaster*; Functional annotation: classification of gene in functional categories; Orthology: single-copy-ortholog (SCO), lineage-restricted (LR), paralog (PAR). Columns 5–8 show the significant genes based on glmLRT (significant genes based on the likelihood ratio test) or glmQLT (significant genes at 5h based on the quasilikelihood ratio test and the normalization used (spAll: all species together, spClade: subset of species, mel: *D. melanogaster*, sec: *D. sechellia*, sim: *D. simulans*, yak: *D. yakuba*); Microarrays: significant genes found in [17] (PAR) and [14] (CvsS); Genome: significant genes found in [19] (SR=Selected Region, SS=Significant Site). (CSV 7 kb)

## Abbreviations

5h: 5 hours after parasitization
50h: 50 hours after parasitization
DEG: Differentially expressed genes
FDR: False discovery rate
glm: Generalized linear model
LR: Lineage restricted
LRT: Likelihood ratio test
PAR: Paralogs
QL: Quasilikelihood ratio test
SCO: Single copy orthologues
