# Supplementary figures and images for: Inter- and intra-species variation in genome-wide gene expression of Drosophila in response to parasitoid wasp attack
Source: BMC Genomics. 2017 Apr 27;18:331. doi: 10.1186/s12864-017-3697-3 (PMC5406980; doi:10.1186/s12864-017-3697-3)

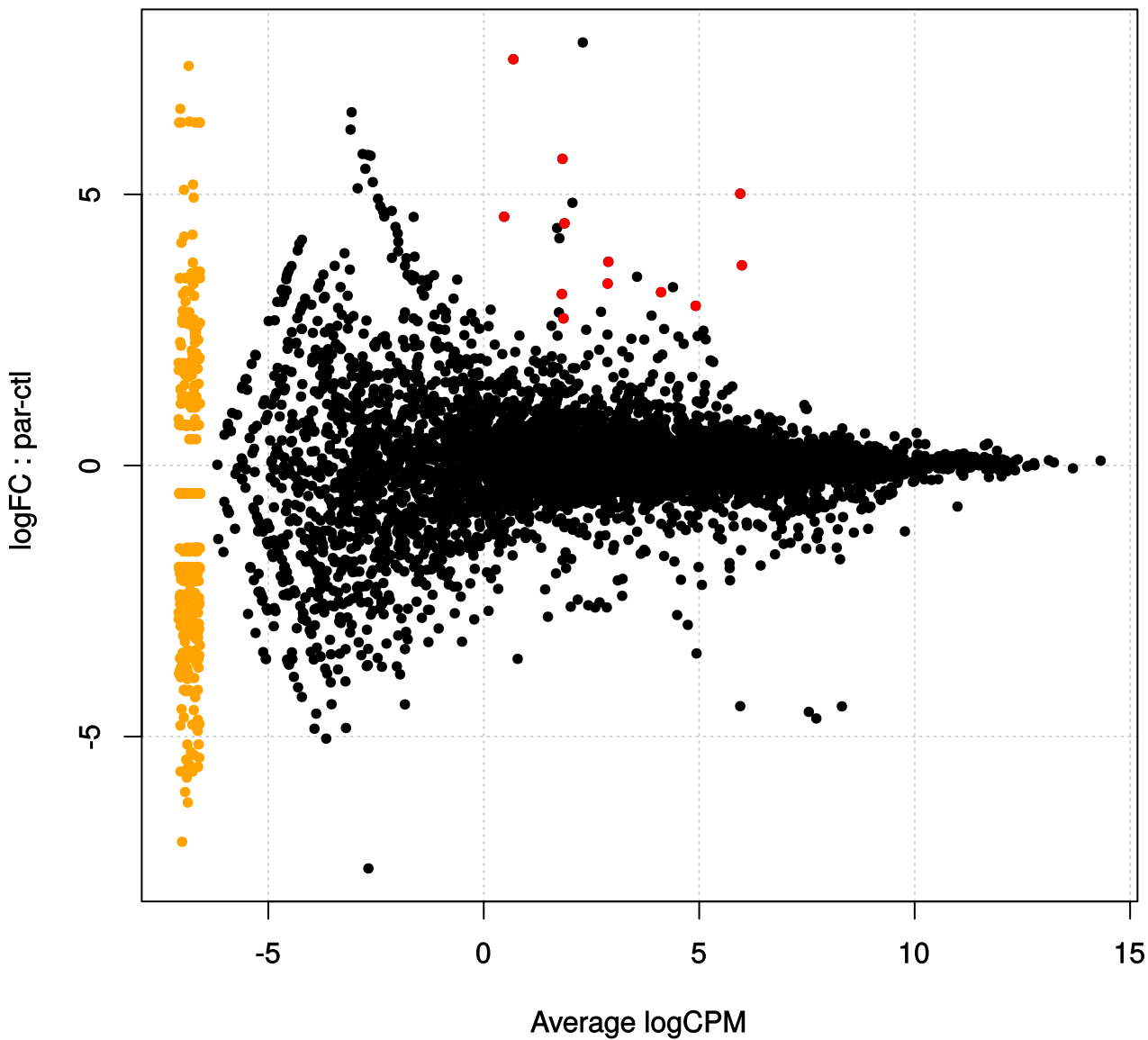

Supplement: Supplementary file 1 — MA Plot for all species 5h. Log-fold change against log-counts per million for all species at 5h. (PDF 338 kb) [file 12864_2017_3697_MOESM1_ESM.pdf]

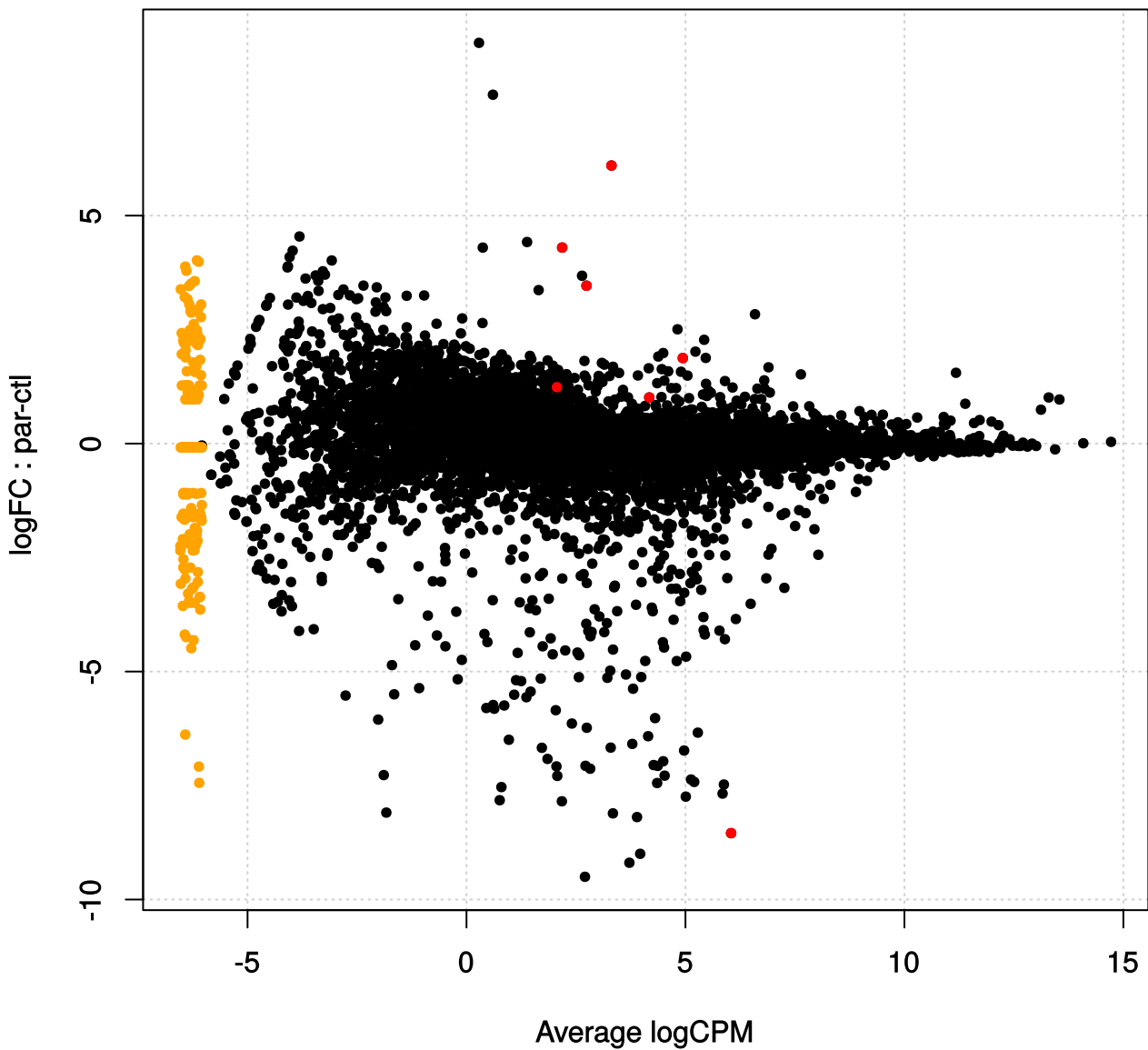

Supplement: Supplementary file 2 — MA Plot for all species 50h. Log-fold change against log-counts per million for all species at 50h. (PDF 337 kb) [file 12864_2017_3697_MOESM2_ESM.pdf]

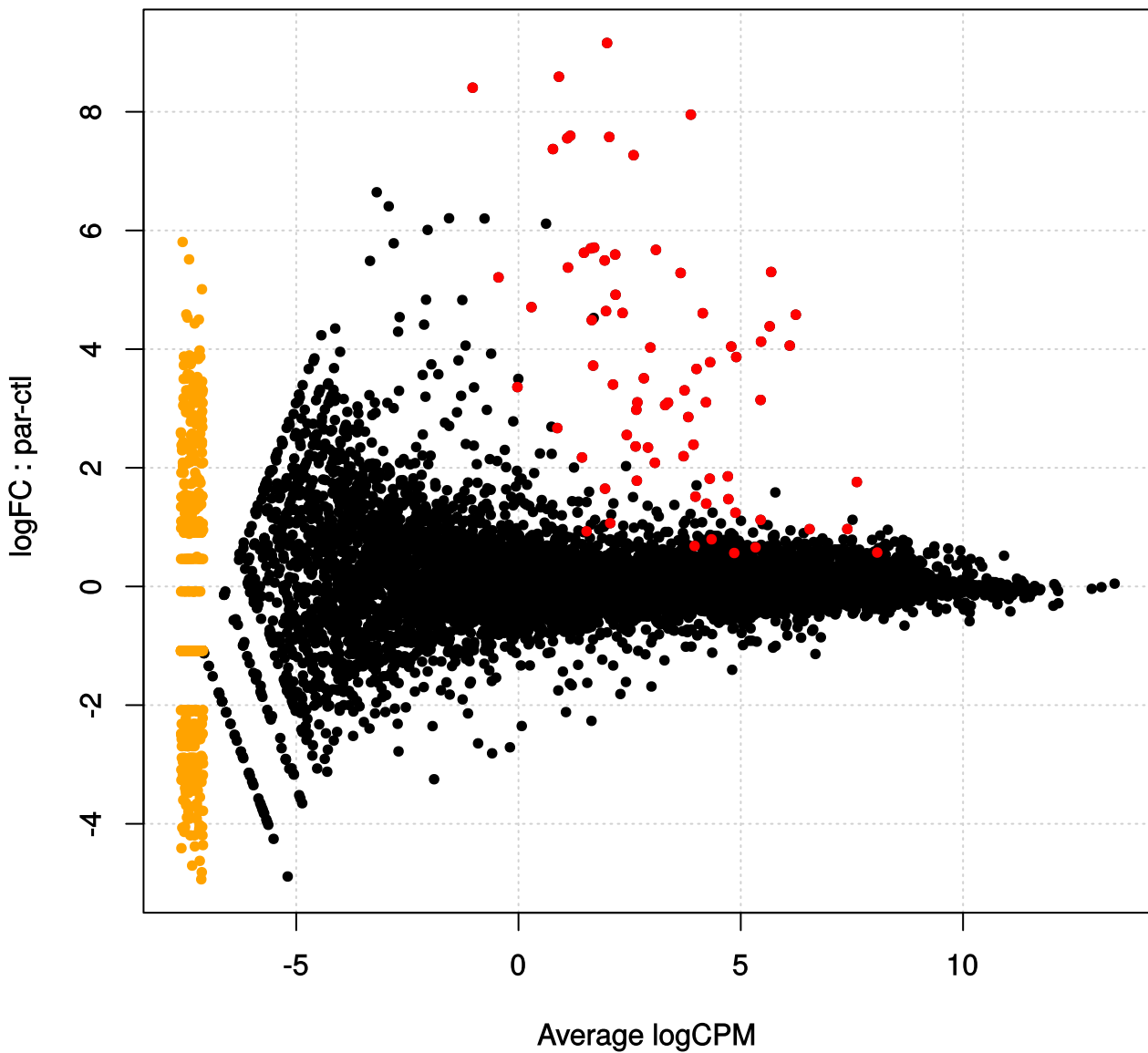

Supplement: Supplementary file 3 — MA Plot for D. melanogaster at 5h. Log-fold change against log-counts per million for D. melanogaster at 5h. (PDF 436 kb) [file 12864_2017_3697_MOESM3_ESM.pdf]

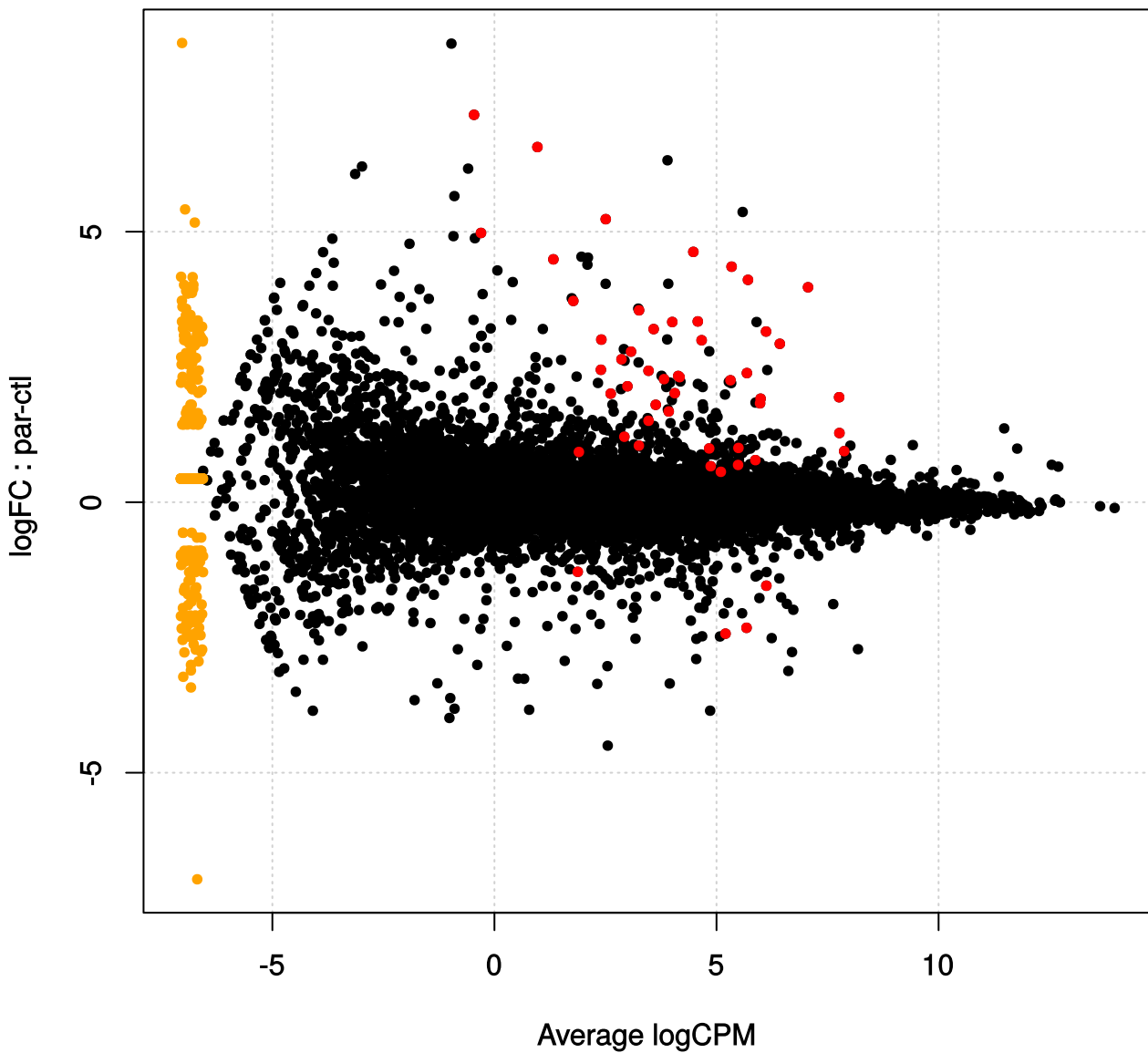

Supplement: Supplementary file 4 — MA Plot for D. melanogaster at 50h. Log-fold change against log-counts per million for D. melanogaster at 50h. (PDF 432 kb) [file 12864_2017_3697_MOESM4_ESM.pdf]

### D.simulans

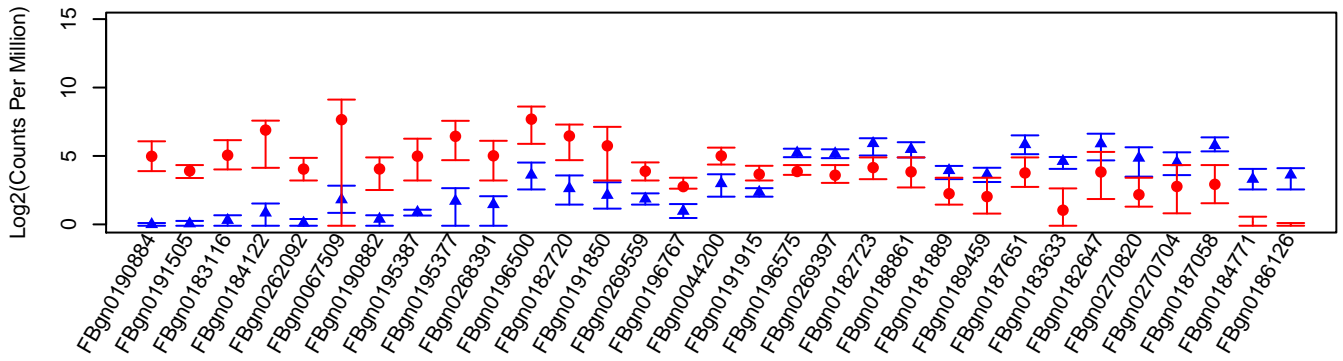

### D.sechellia

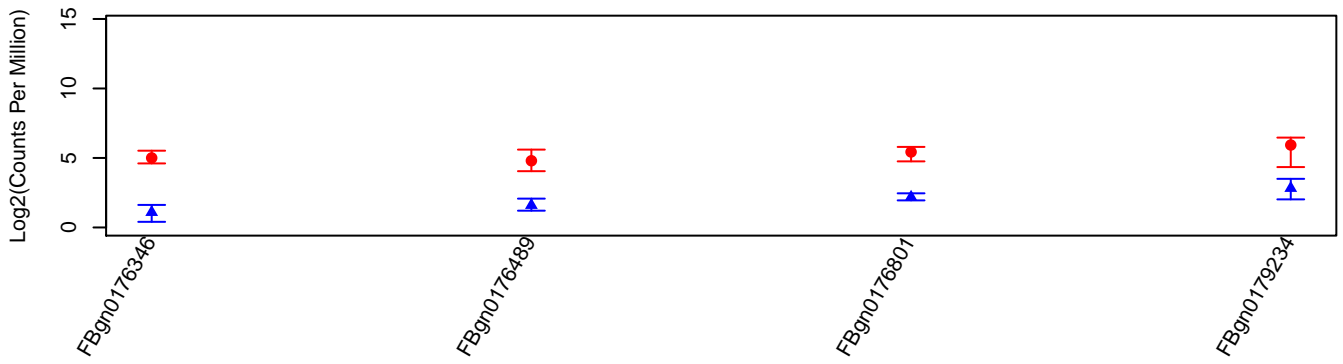

### D.yakuba

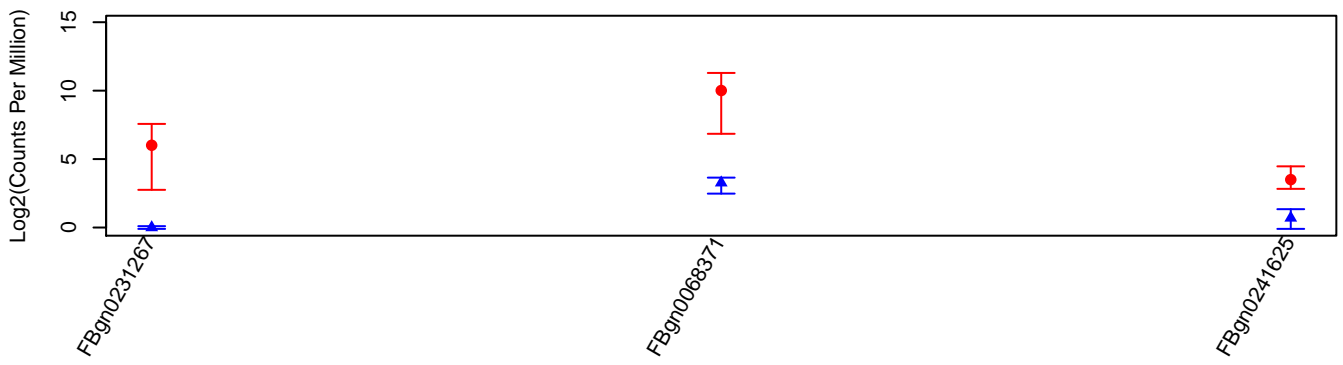

Supplement: Supplementary file 7 — CPM Plot for D. simulans, D. sechellia, D. yakuba at 5h. Log2 counts per million of control (blue triangles) and parasitized (red circles) for D. simulans, D. sechellia, D. yakuba at 5h. (PDF 9 kb) [file 12864_2017_3697_MOESM7_ESM.pdf]

D.simulans

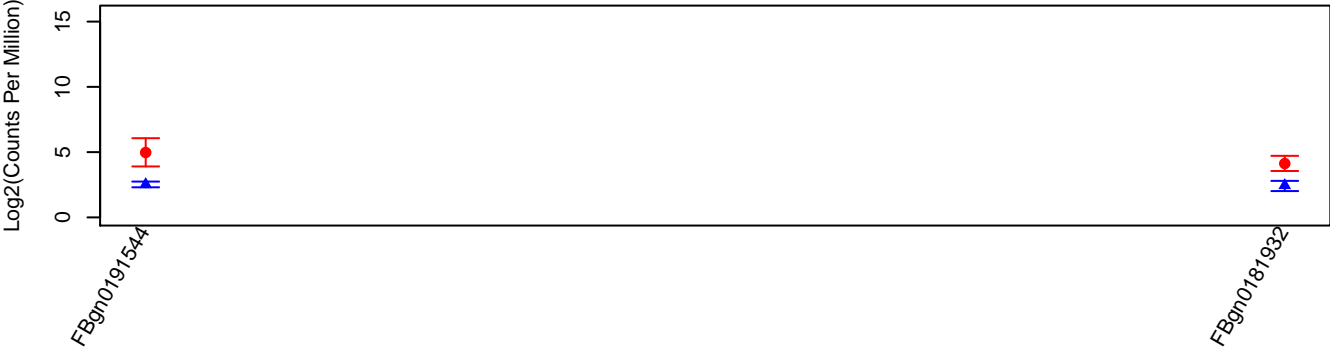

Supplement: Supplementary file 8 — CPM Plot for D. simulans at 50h. Log2 counts per million of control (blue triangles) and parasitized (red circles) for D. simulans at 5h. (PDF 5 kb) [file 12864_2017_3697_MOESM8_ESM.pdf]

# MDS all species 5h

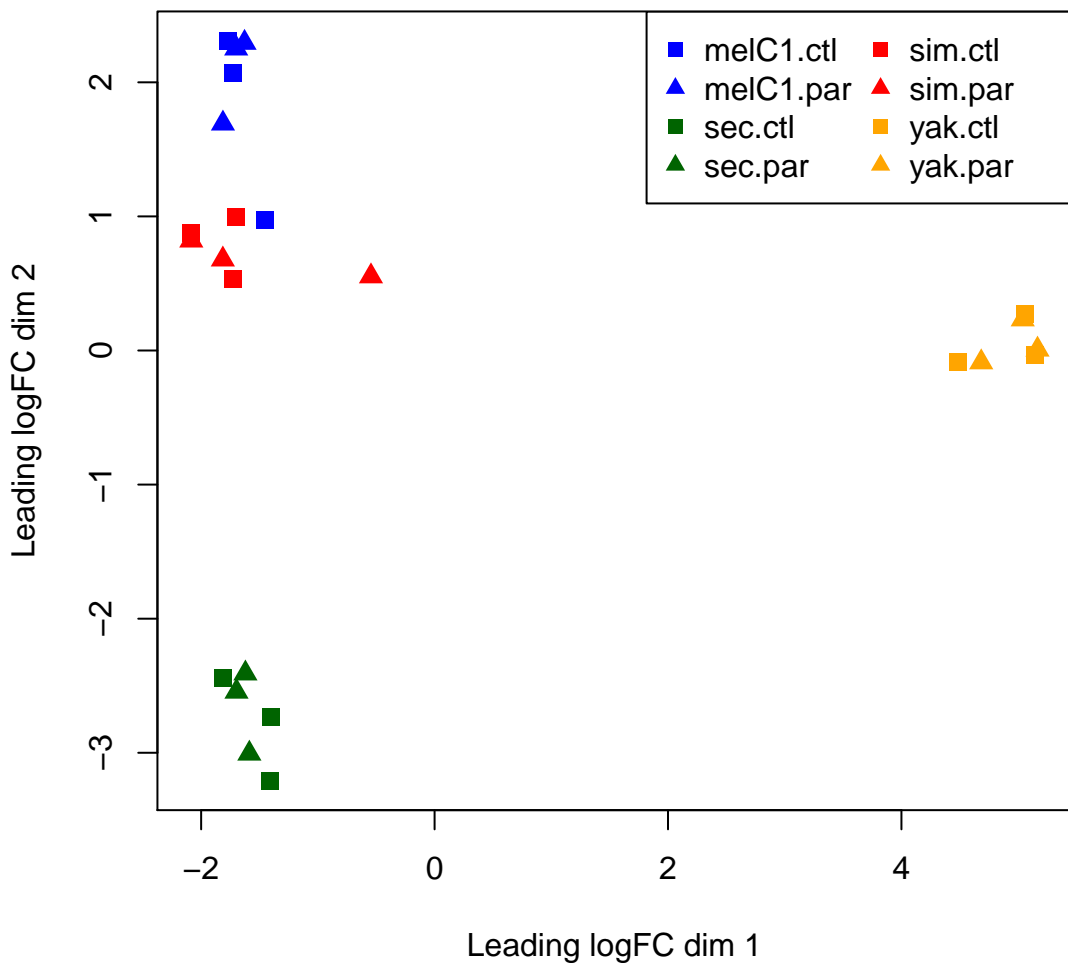

Supplement: Supplementary file 9 — MDS Plot for all species 5h. Multidimensional scaling plot showing gene expression distances for all species at 5h. (PDF 5 kb) [file 12864_2017_3697_MOESM9_ESM.pdf]

# MDS all species 50h

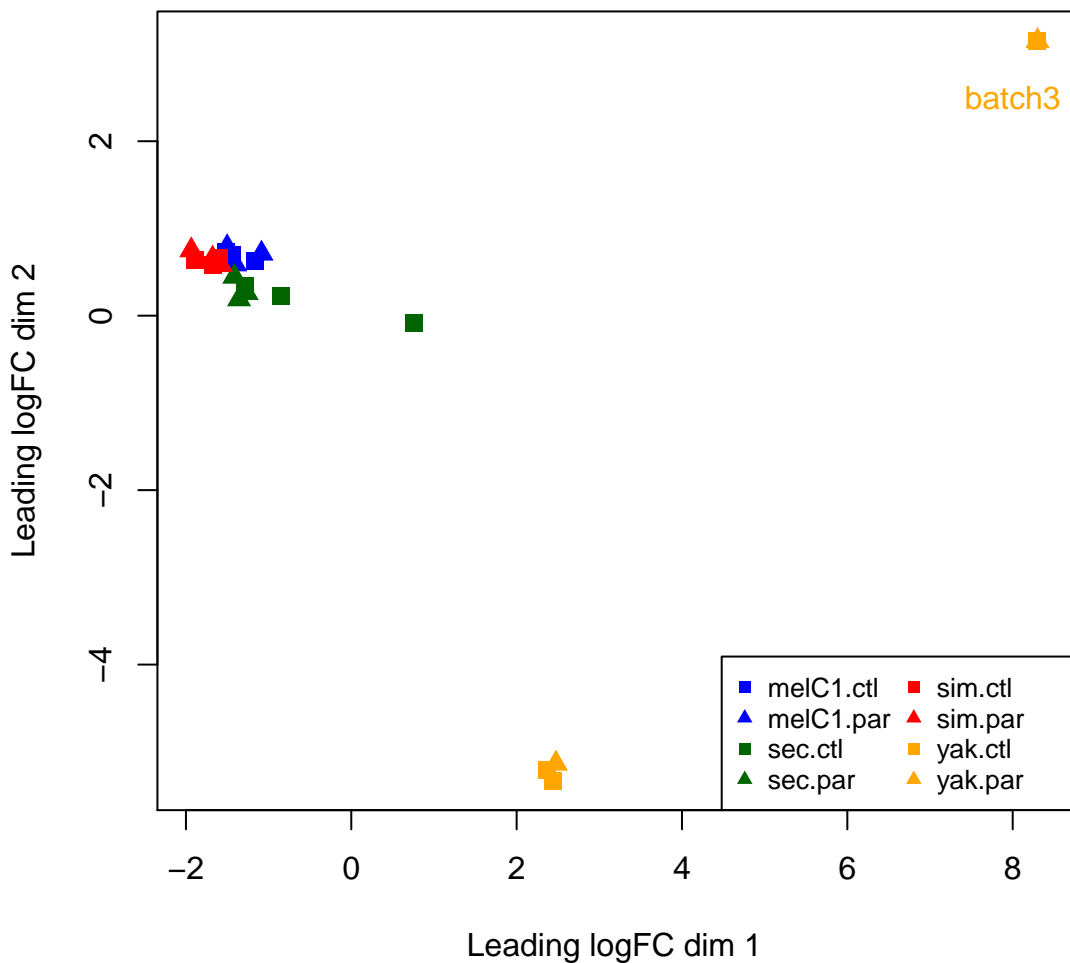

Supplement: Supplementary file 10 — MDS Plot for all species 50h. Multidimensional scaling plot showing gene expression distances for all species at 50h. (PDF 5 kb) [file 12864_2017_3697_MOESM10_ESM.pdf]

# MDS D. melanogaster 5h

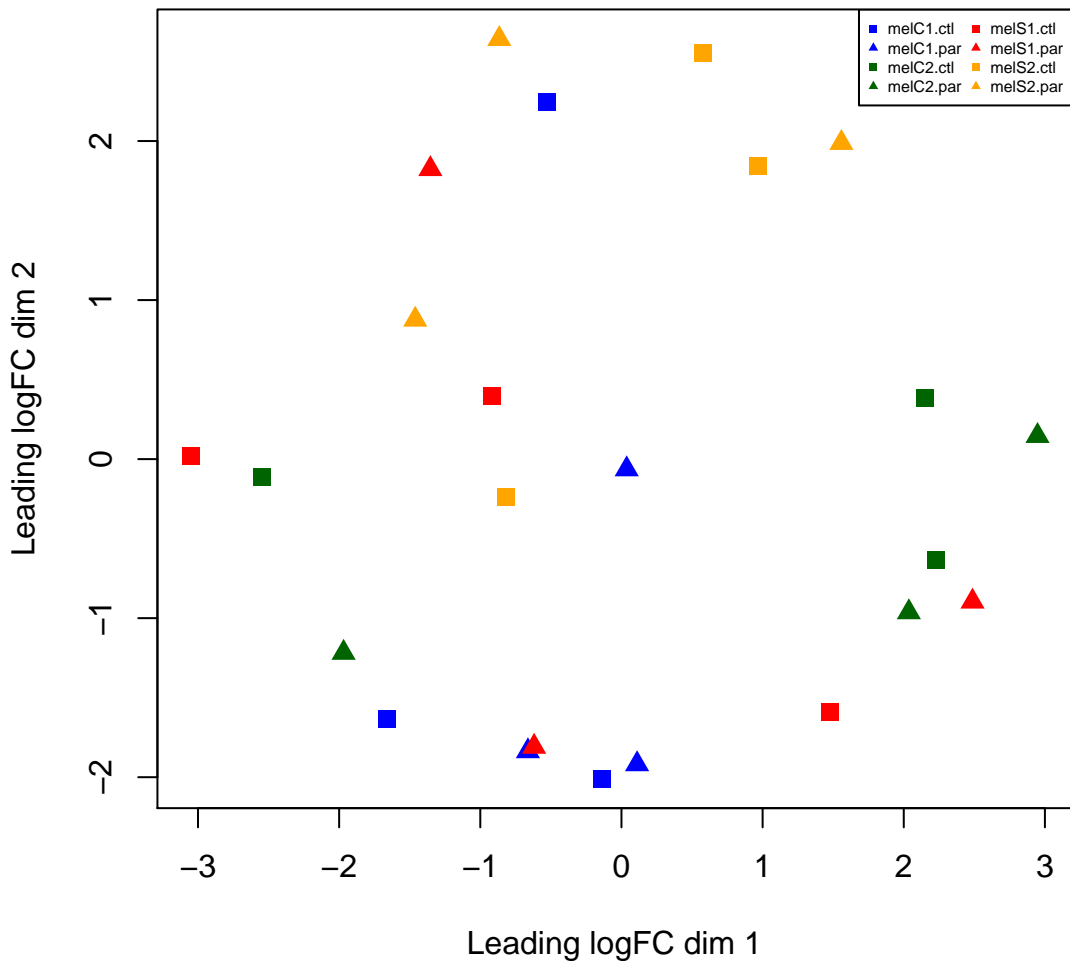

Supplement: Supplementary file 11 — MDS Plot for D. melanogaster 5h. Multidimensional scaling plot showing gene expression distances for D. melanogaster 5h. (PDF 5 kb) [file 12864_2017_3697_MOESM11_ESM.pdf]

# MDS D. melanogaster 50h

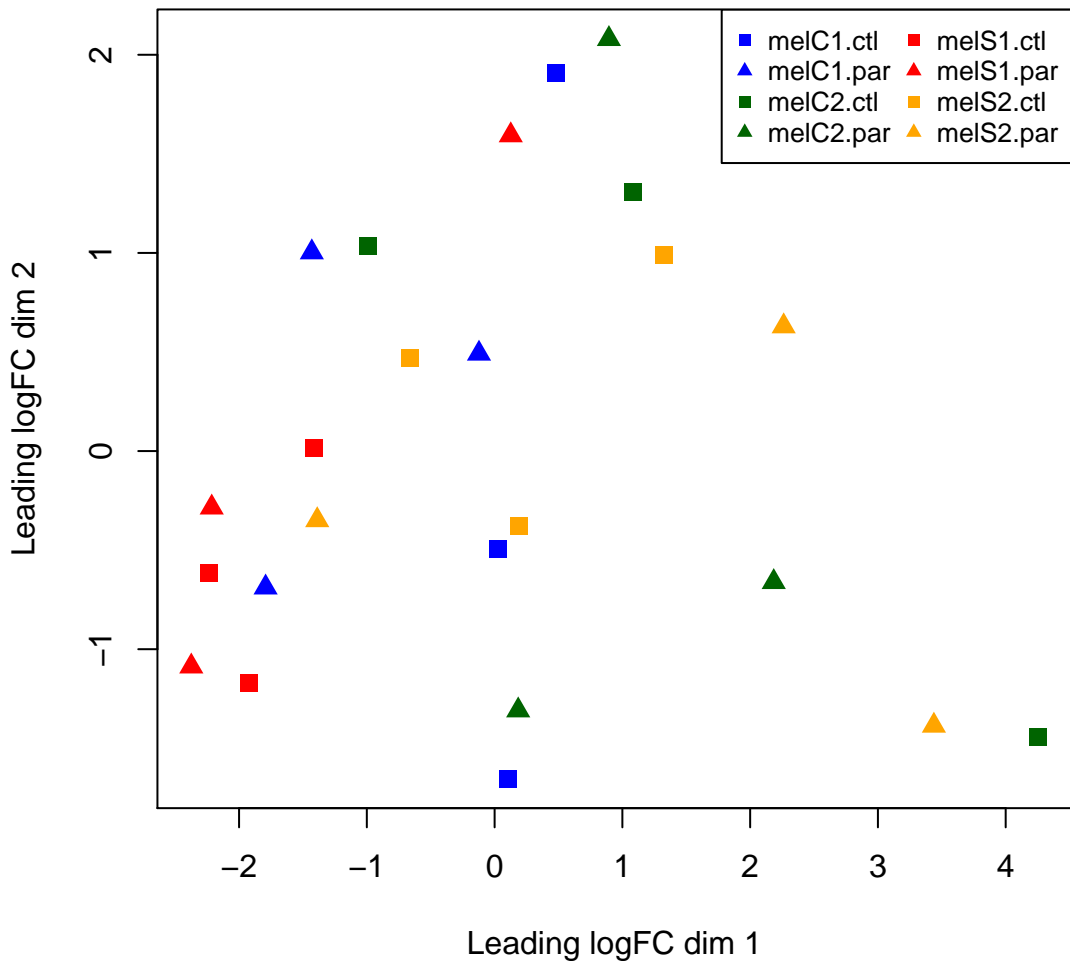

Supplement: Supplementary file 12 — MDS Plot for D. melanogaster 50h. Multidimensional scaling plot showing gene expression distances for D. melanogaster 50h. (PDF 5 kb) [file 12864_2017_3697_MOESM12_ESM.pdf]

FBgn0040734 (CG15065)

ctl

par

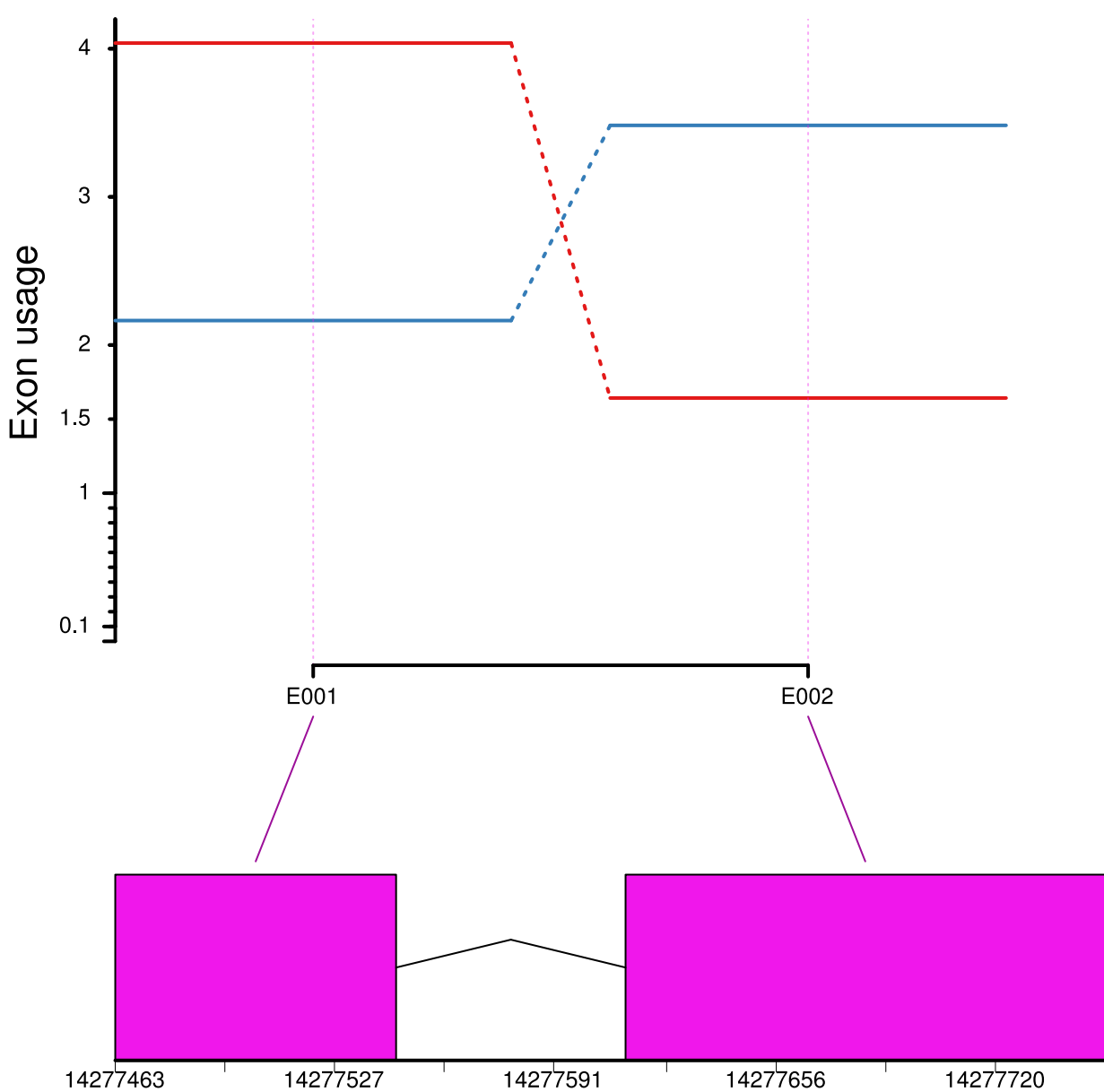

Supplement: Supplementary file 14 — Differential exon usage of CG15065 “par”. Fitted expression value of CG15065 showing in pink the exon with significant differential expression. (PDF 18 kb) [file 12864_2017_3697_MOESM14_ESM.pdf]

# FBgn0041182 (Tep2)

ctl

par

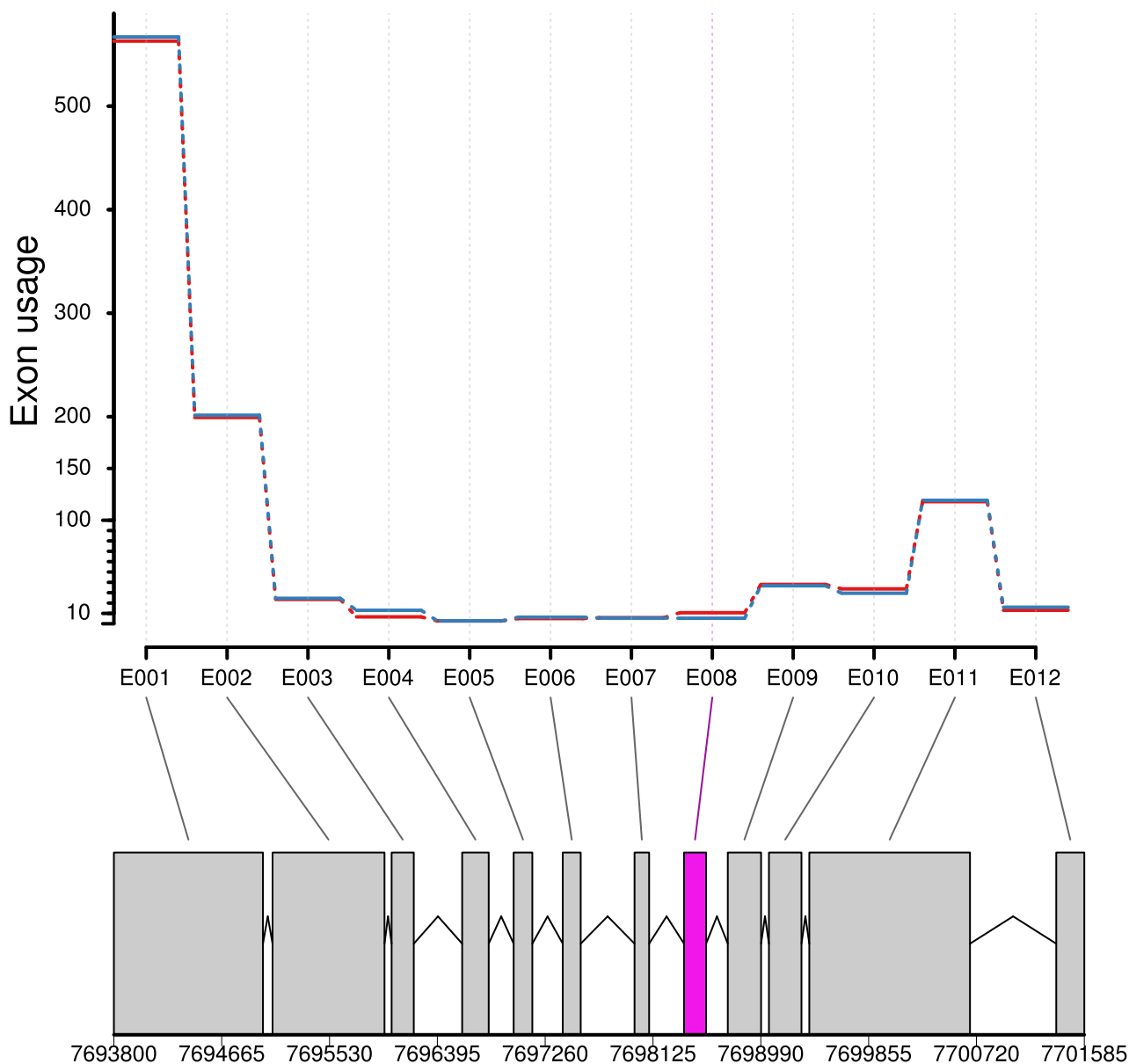

Supplement: Supplementary file 15 — Differential exon usage Tep2 “par”. Fitted expression value of Tep2 showing in pink the exon with significant differential expression. (PDF 37 kb) [file 12864_2017_3697_MOESM15_ESM.pdf]

FBgn0041182 (Tep2)

C.ctl C.par S.ctl S.par

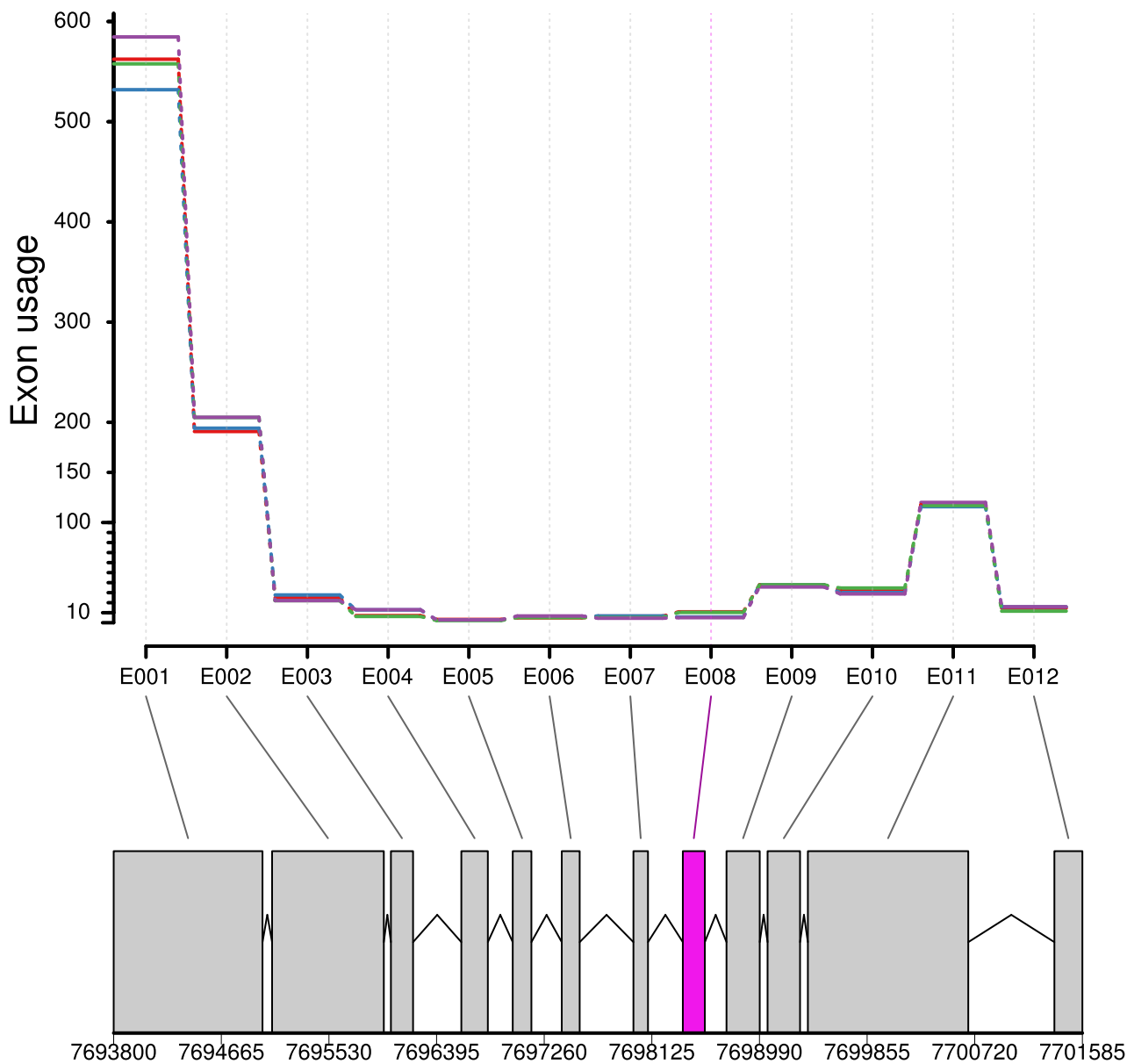

Supplement: Supplementary file 16 — Differential exon usage Tep2 “int”. Fitted expression value of Tep2 showing in pink the exon with significant differential expression. (PDF 39 kb) [file 12864_2017_3697_MOESM16_ESM.pdf]

FBgn0262607 (CG43133)

ctl

par

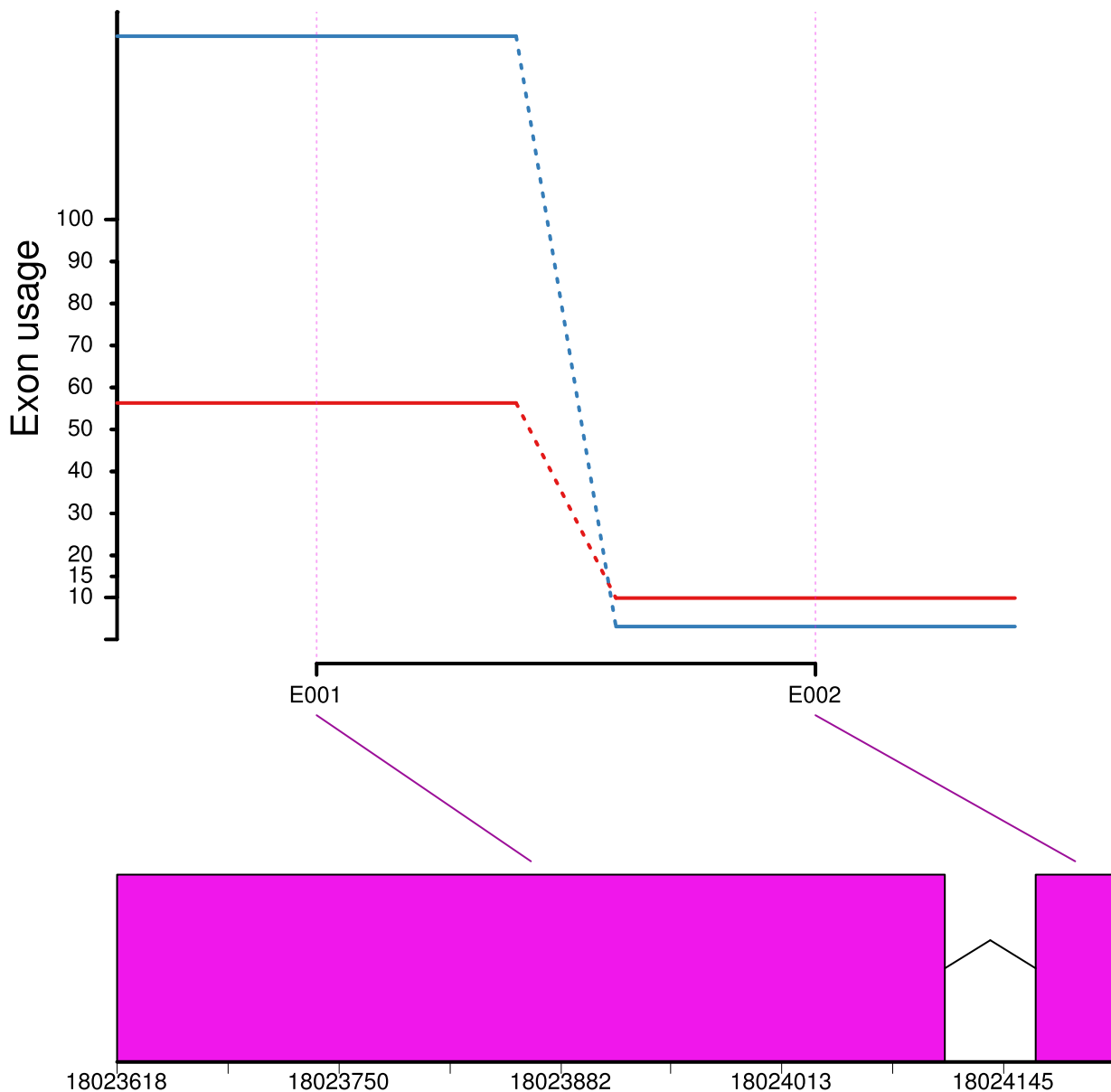

Supplement: Supplementary file 17 — Differential exon usage CG43133 “par”. Fitted expression value of CG43133 showing in pink the exon with significant differential expression. (PDF 22 kb) [file 12864_2017_3697_MOESM17_ESM.pdf]

FBgn0262607 (CG43133)

C.ctl C.par S.ctl S.par

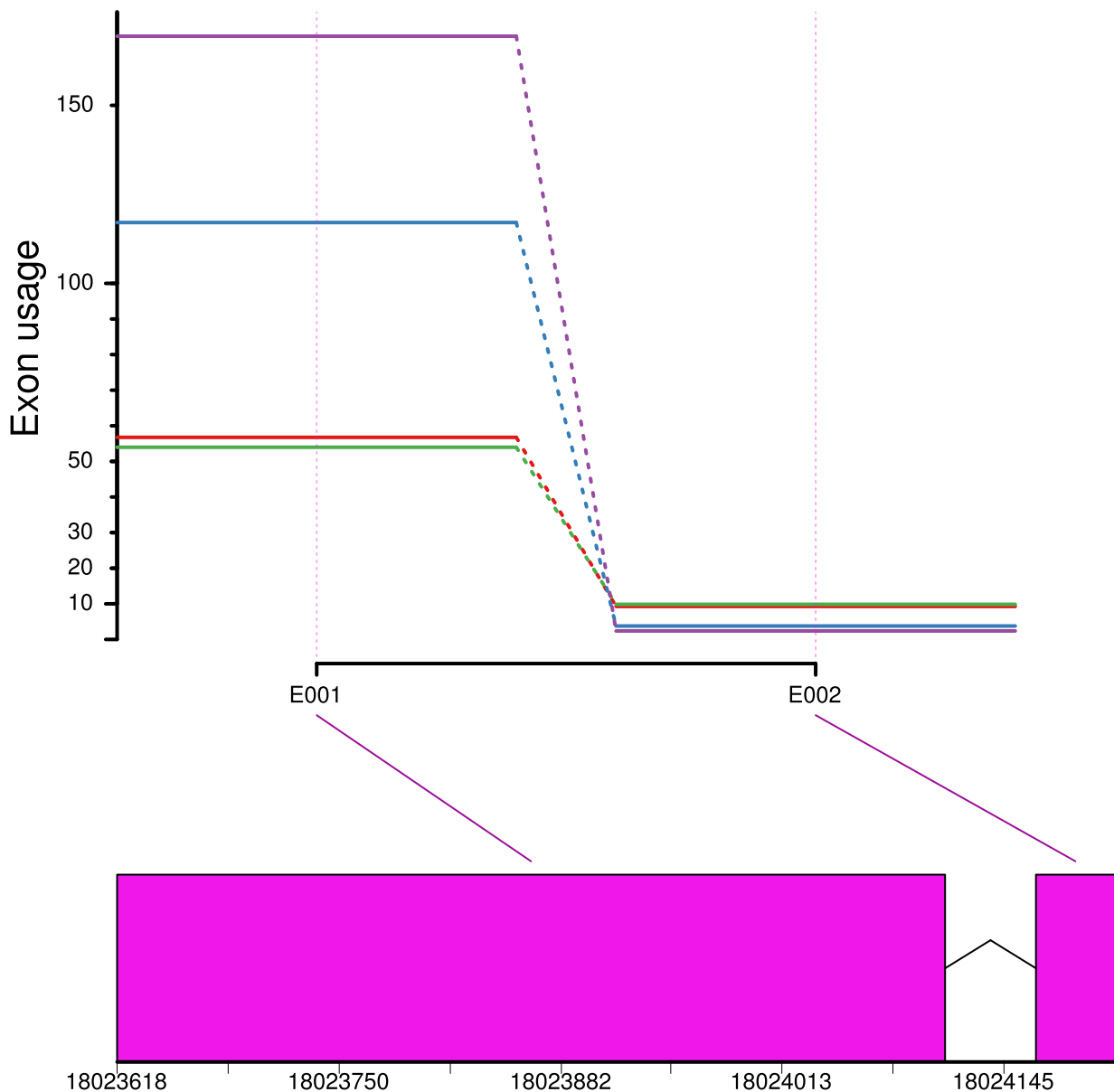

Supplement: Supplementary file 18 — Differential exon usage CG43133 “int”. Fitted expression value of CG43133 showing in pink the exon with significant differential expression. (PDF 23 kb) [file 12864_2017_3697_MOESM18_ESM.pdf]

FBgn0038247 (fok)

C

S

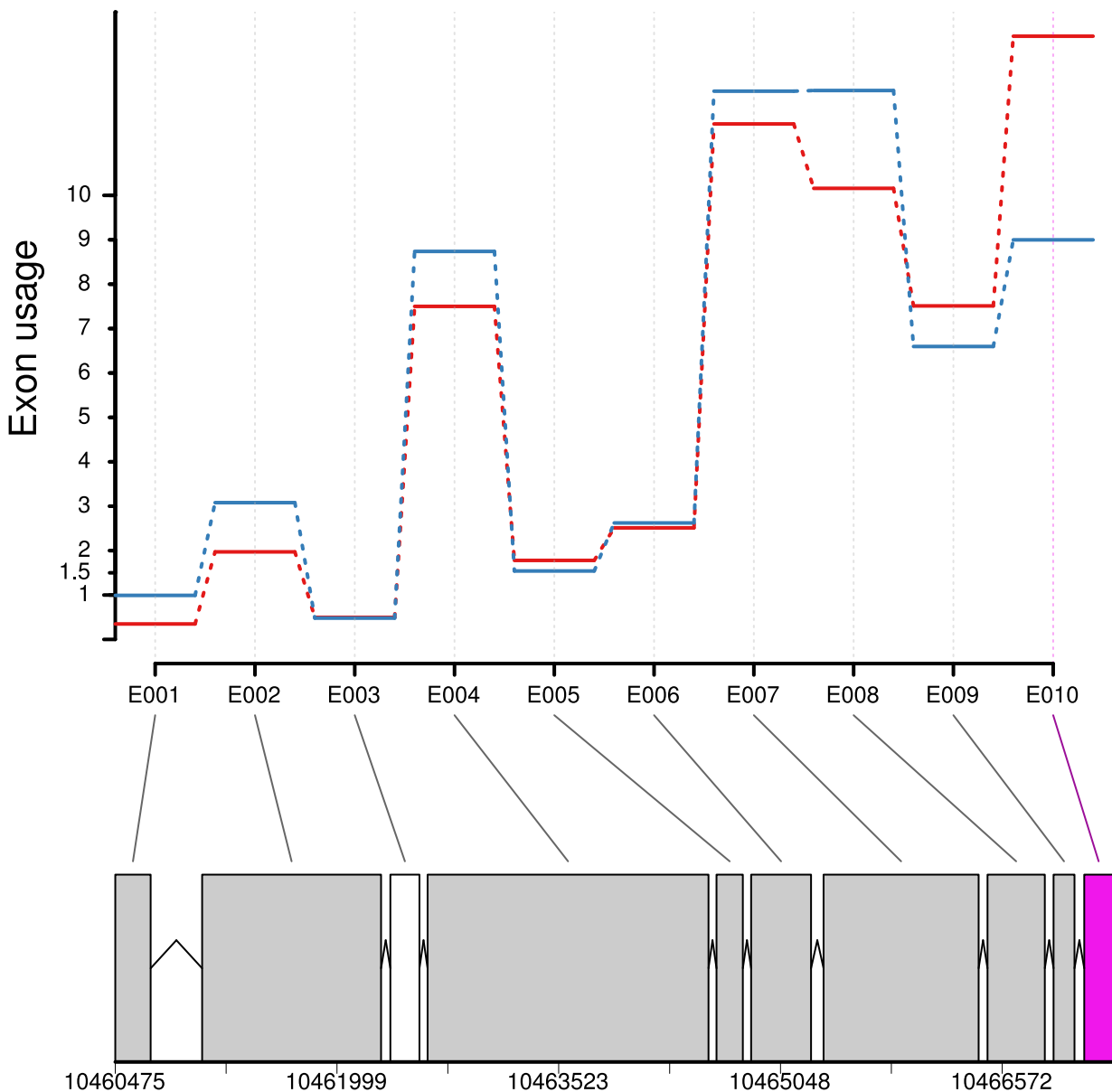

Supplement: Supplementary file 19 — Differential exon usage of fok “sel”. Fitted expression value of fok showing in pink the exon with significant differential expression. (PDF 28 kb) [file 12864_2017_3697_MOESM19_ESM.pdf]

FBgn0014469 (CG2060)

C

S

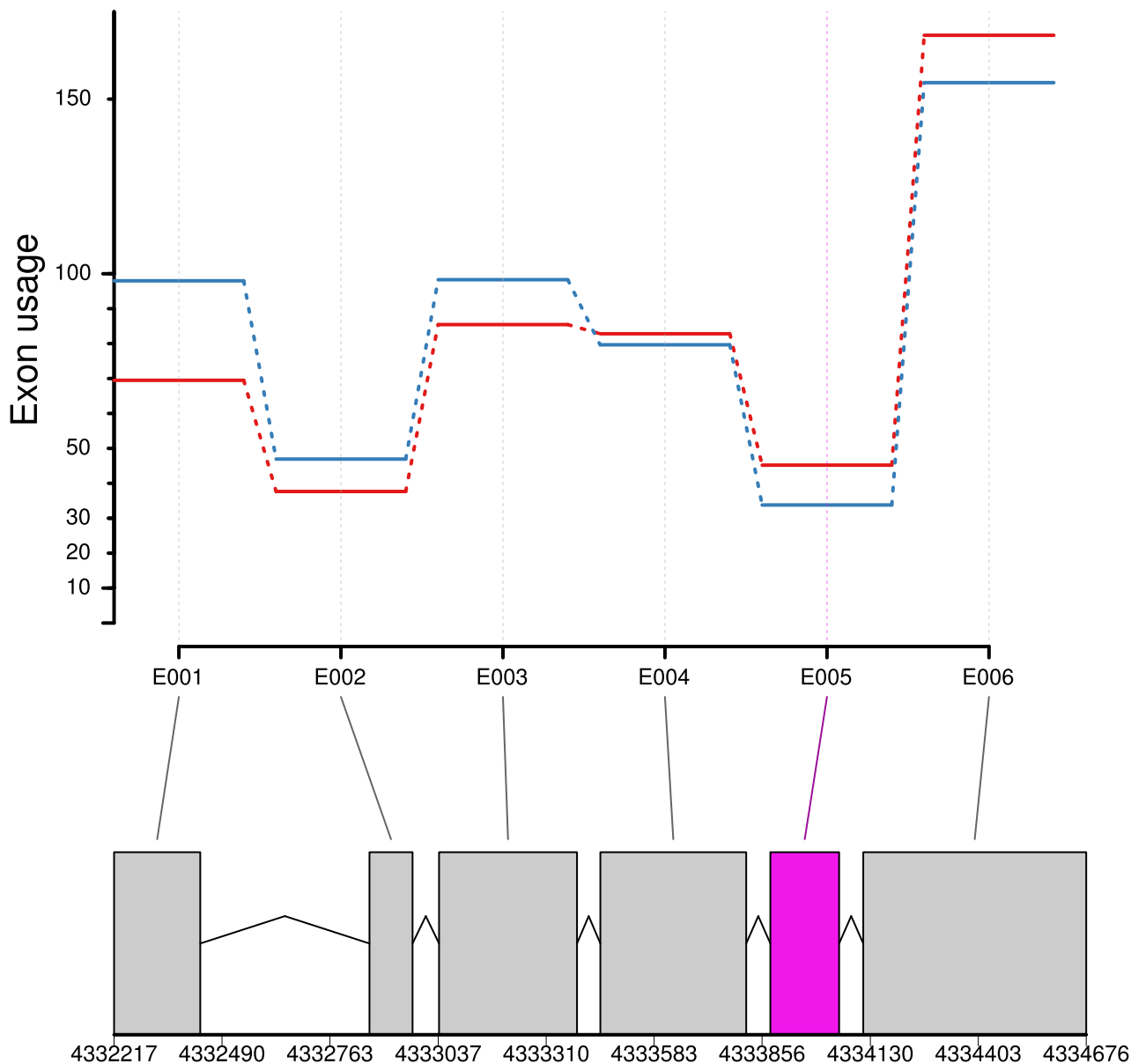

Supplement: Supplementary file 20 — Differential exon usage CG2060 “sel”. Fitted expression value of CG2060 showing in pink the exon with significant differential expression. (PDF 30 kb) [file 12864_2017_3697_MOESM20_ESM.pdf]

FBgn0263773 (CG3389)

C.ctl C.par S.ctl S.par

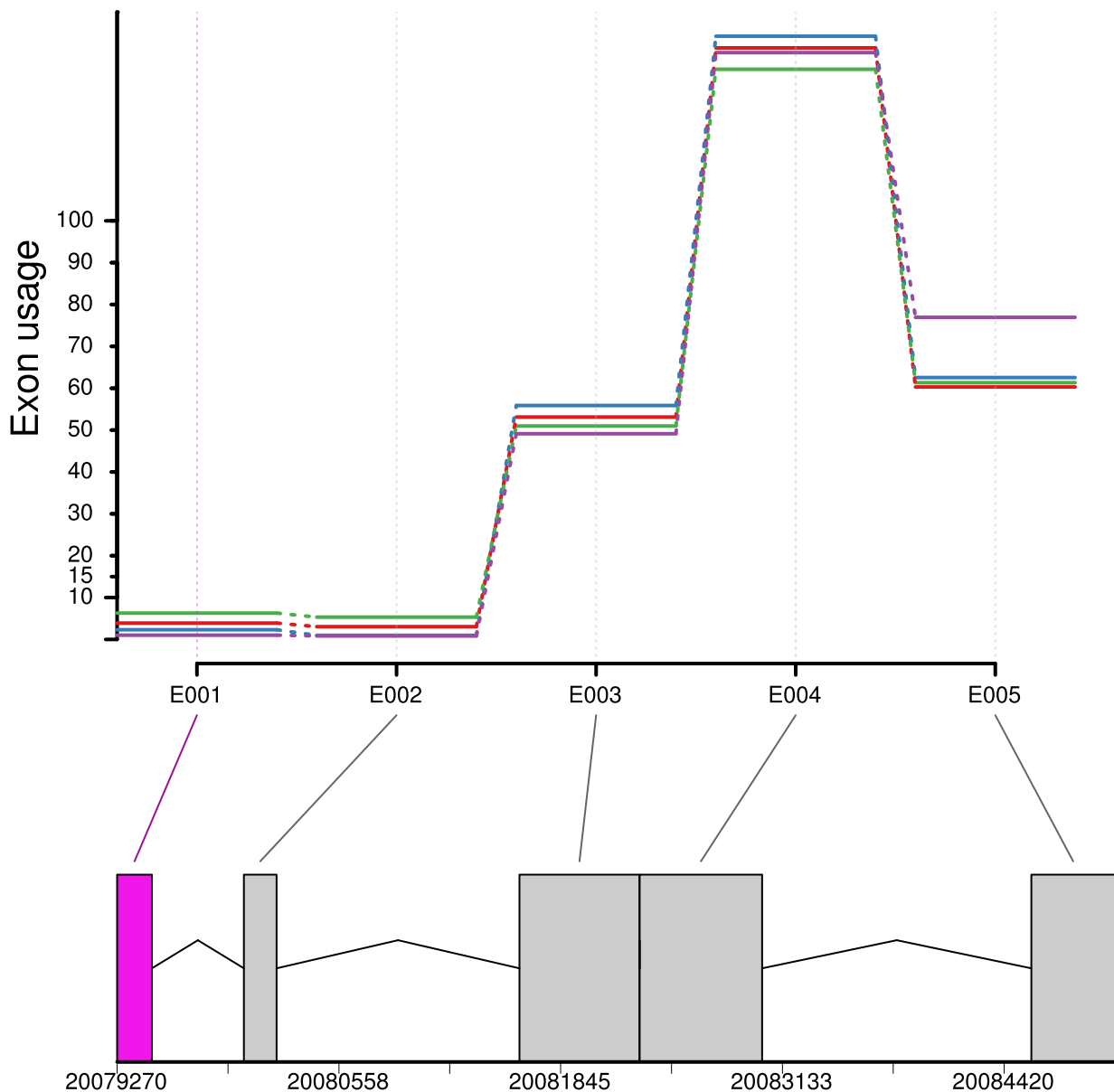

Supplement: Supplementary file 21 — Differential exon usage CG3389 “int”. Fitted expression value of CG3389 showing in pink the exon with significant differential expression. (PDF 28 kb) [file 12864_2017_3697_MOESM21_ESM.pdf]

FBgn0262869 (CG33332)

C.ctl C.par S.ctl S.par

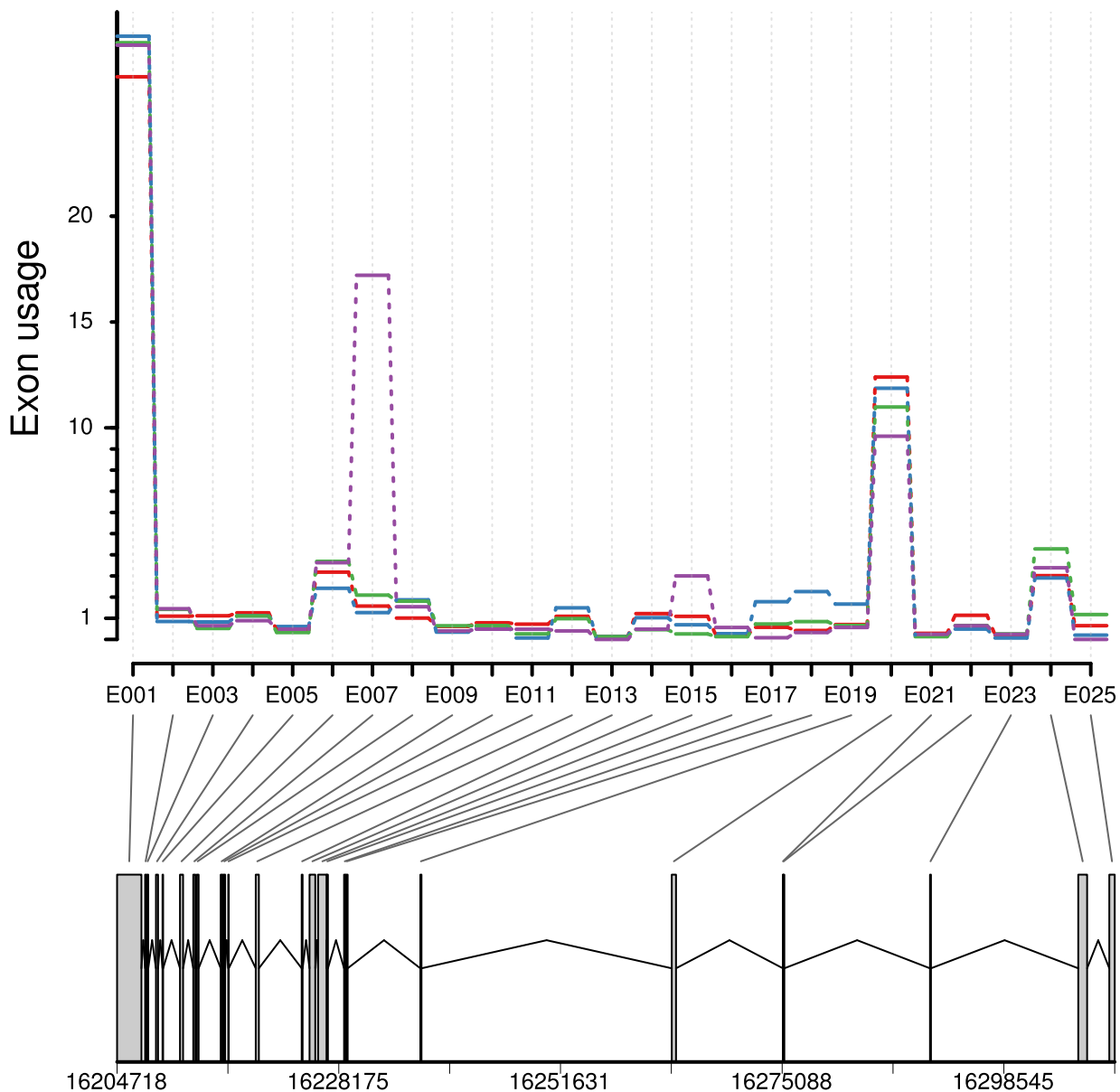

Supplement: Supplementary file 22 — Differential exon usage CG33332 “int”. Fitted expression value of CG33332 showing in pink the exon with significant differential expression. (PDF 40 kb) [file 12864_2017_3697_MOESM22_ESM.pdf]
