# Supplementary material for: Inter- and intra-species variation in genome-wide gene expression of Drosophila in response to parasitoid wasp attack
Source: BMC Genomics. 2017 Apr 27;18:331. doi: 10.1186/s12864-017-3697-3 (PMC5406980; doi:10.1186/s12864-017-3697-3)
Supplement: Supplementary file 5 — CPM Plot for D. melanogaster at 5h. Log2 counts per million of control (blue triangles) and parasitized (red circles) for D. melanogaster at 5h. (PDF 39 kb) [file 12864_2017_3697_MOESM5_ESM.pdf]

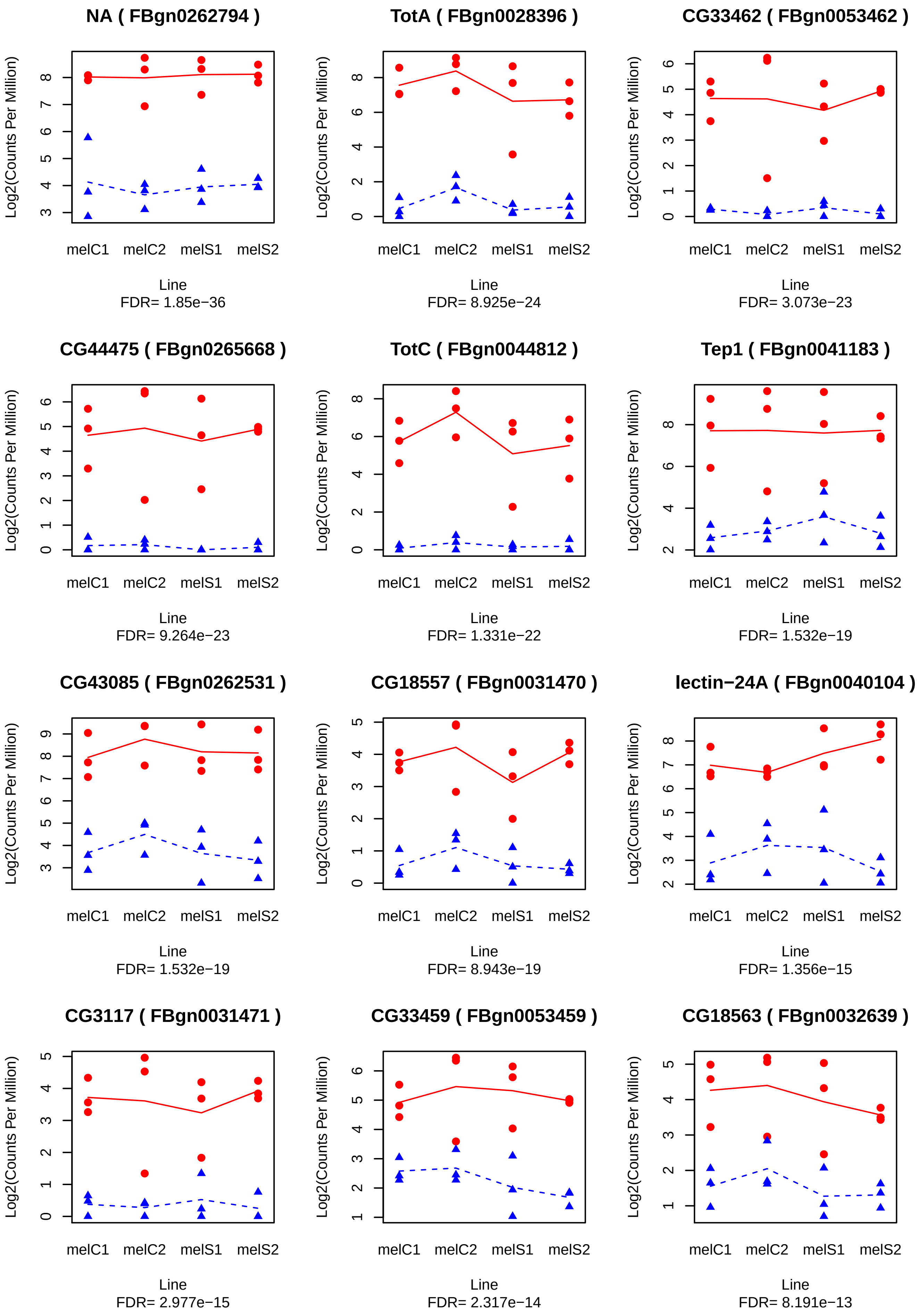
NA (FBgn0262794)
Log2(Counts Per Million)
melC1 melC2 melS1 melS2
Line
FDR= 1.85e−36
TotA (FBgn0028396)
Log2(Counts Per Million)
melC1 melC2 melS1 melS2
Line
FDR= 8.925e−24
CG33462 (FBgn0053462)
Log2(Counts Per Million)
melC1 melC2 melS1 melS2
Line
FDR= 3.073e−23
CG44475 (FBgn0265668)
Log2(Counts Per Million)
melC1 melC2 melS1 melS2
Line
FDR= 9.264e−23
TotC (FBgn0044812)
Log2(Counts Per Million)
melC1 melC2 melS1 melS2
Line
FDR= 1.331e−22
Tep1 (FBgn0041183)
Log2(Counts Per Million)
melC1 melC2 melS1 melS2
Line
FDR= 1.532e−19
CG43085 (FBgn0262531)
Log2(Counts Per Million)
melC1 melC2 melS1 melS2
Line
FDR= 1.532e−19
CG18557 (FBgn0031470)
Log2(Counts Per Million)
melC1 melC2 melS1 melS2
Line
FDR= 8.943e−19
lectin−24A (FBgn0040104)
Log2(Counts Per Million)
melC1 melC2 melS1 melS2
Line
FDR= 1.356e−15
CG3117 (FBgn0031471)
Log2(Counts Per Million)
melC1 melC2 melS1 melS2
Line
FDR= 2.977e−15
CG33459 (FBgn0053459)
Log2(Counts Per Million)
melC1 melC2 melS1 melS2
Line
FDR= 2.317e−14
CG18563 (FBgn0032639)
Log2(Counts Per Million)
melC1 melC2 melS1 melS2
Line
FDR= 8.191e−13

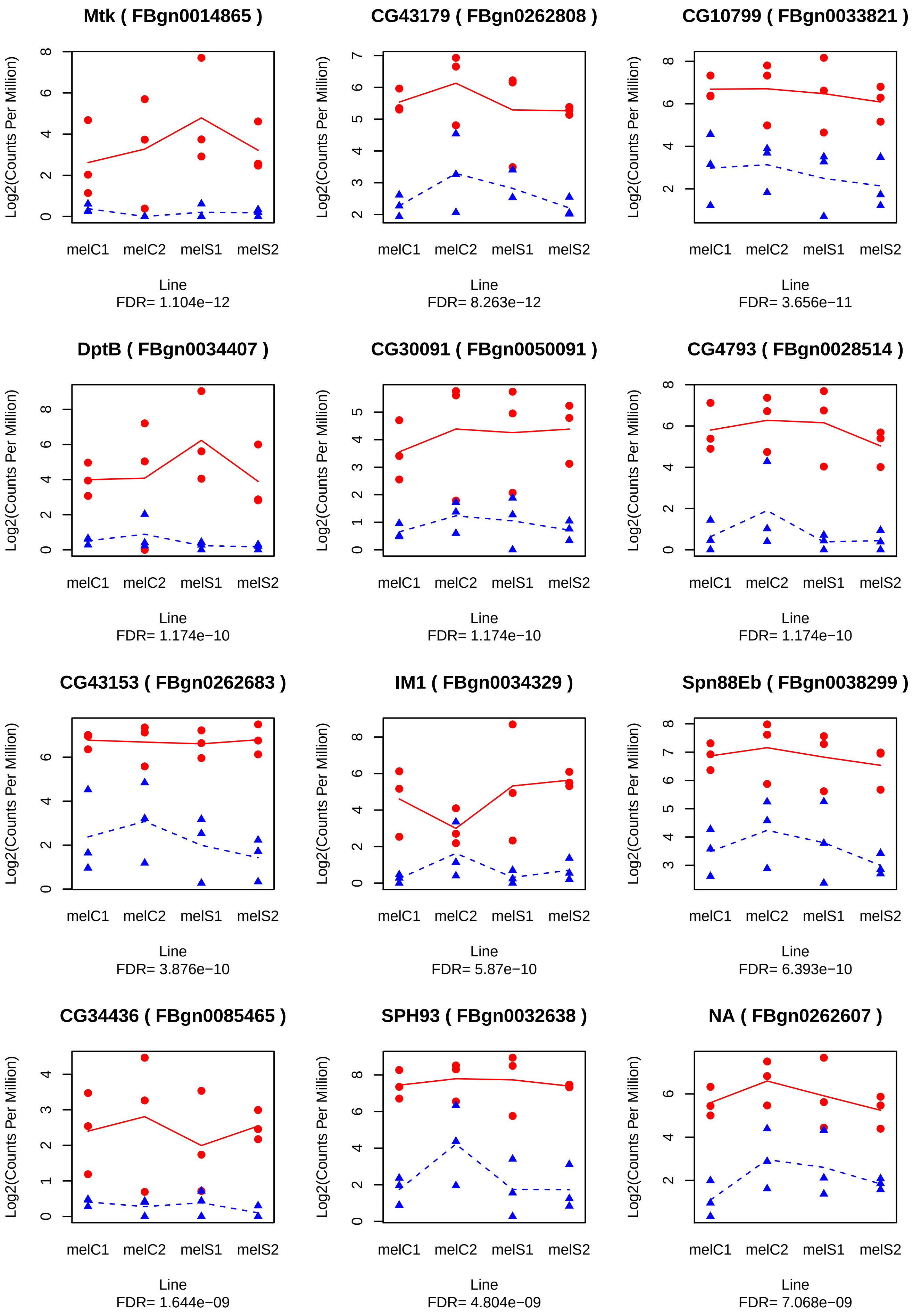
Mtk (FBgn0014865)
Log2(Counts Per Million)
melC1 melC2 melS1 melS2
Line
FDR= 1.104e−12
CG43179 (FBgn0262808)
Log2(Counts Per Million)
melC1 melC2 melS1 melS2
Line
FDR= 8.263e−12
CG10799 (FBgn0033821)
Log2(Counts Per Million)
melC1 melC2 melS1 melS2
Line
FDR= 3.656e−11
DptB (FBgn0034407)
Log2(Counts Per Million)
melC1 melC2 melS1 melS2
Line
FDR= 1.174e−10
CG30091 (FBgn0050091)
Log2(Counts Per Million)
melC1 melC2 melS1 melS2
Line
FDR= 1.174e−10
CG4793 (FBgn0028514)
Log2(Counts Per Million)
melC1 melC2 melS1 melS2
Line
FDR= 1.174e−10
CG43153 (FBgn0262683)
Log2(Counts Per Million)
melC1 melC2 melS1 melS2
Line
FDR= 3.876e−10
IM1 (FBgn0034329)
Log2(Counts Per Million)
melC1 melC2 melS1 melS2
Line
FDR= 5.87e−10
Spn88Eb (FBgn0038299)
Log2(Counts Per Million)
melC1 melC2 melS1 melS2
Line
FDR= 6.393e−10
CG34436 (FBgn0085465)
Log2(Counts Per Million)
melC1 melC2 melS1 melS2
Line
FDR= 1.644e−09
SPH93 (FBgn0032638)
Log2(Counts Per Million)
melC1 melC2 melS1 melS2
Line
FDR= 4.804e−09
NA (FBgn0262607)
Log2(Counts Per Million)
melC1 melC2 melS1 melS2
Line
FDR= 7.068e−09

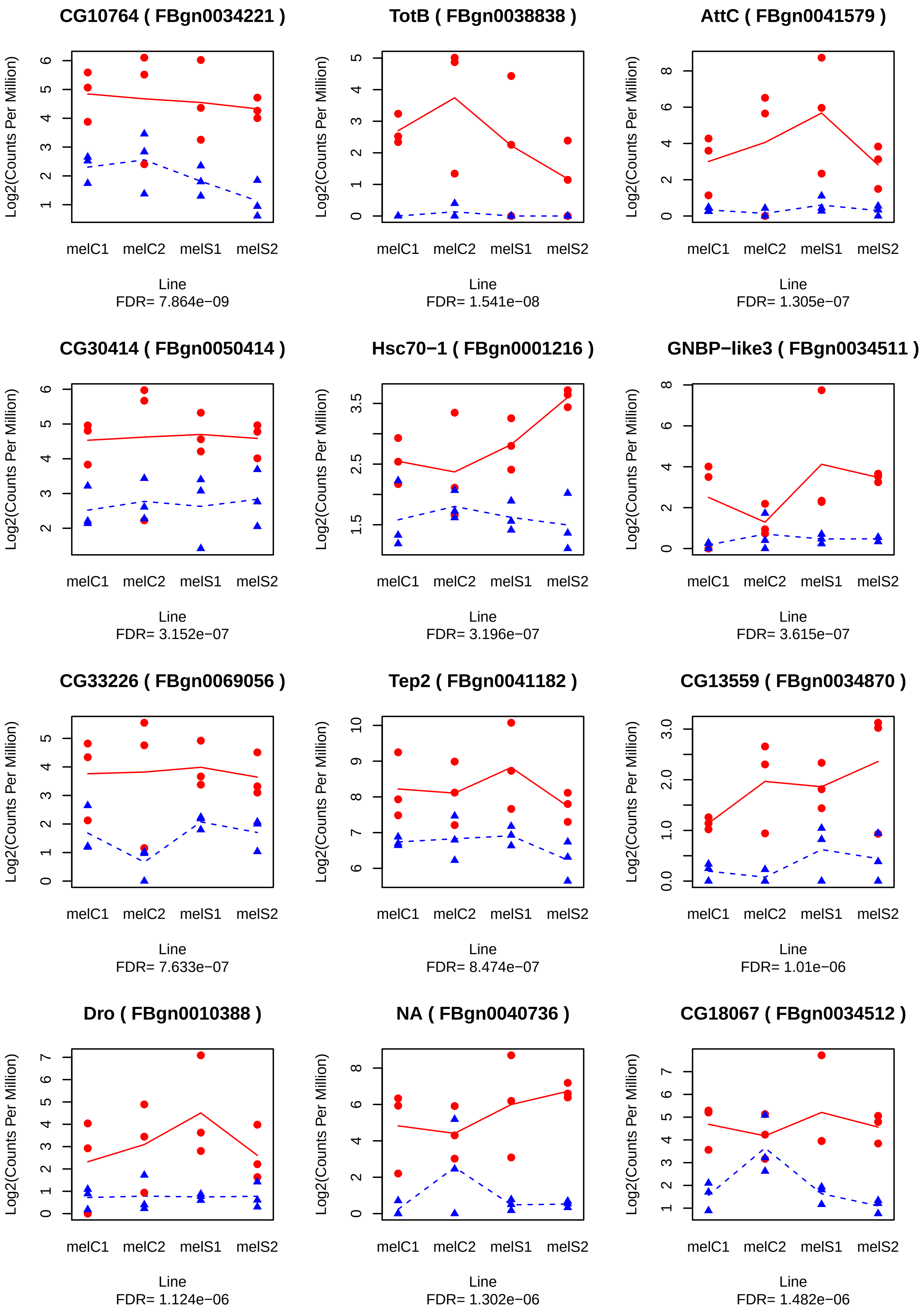
CG10764 (FBgn0034221)
Log2(Counts Per Million)
melC1 melC2 melS1 melS2
Line
FDR= 7.864e−09
TotB (FBgn0038838)
Log2(Counts Per Million)
melC1 melC2 melS1 melS2
Line
FDR= 1.541e−08
AttC (FBgn0041579)
Log2(Counts Per Million)
melC1 melC2 melS1 melS2
Line
FDR= 1.305e−07
CG30414 (FBgn0050414)
Log2(Counts Per Million)
melC1 melC2 melS1 melS2
Line
FDR= 3.152e−07
Hsc70−1 (FBgn0001216)
Log2(Counts Per Million)
melC1 melC2 melS1 melS2
Line
FDR= 3.196e−07
GNBP−like3 (FBgn0034511)
Log2(Counts Per Million)
melC1 melC2 melS1 melS2
Line
FDR= 3.615e−07
CG33226 (FBgn0069056)
Log2(Counts Per Million)
melC1 melC2 melS1 melS2
Line
FDR= 7.633e−07
Tep2 (FBgn0041182)
Log2(Counts Per Million)
melC1 melC2 melS1 melS2
Line
FDR= 8.474e−07
CG13559 (FBgn0034870)
Log2(Counts Per Million)
melC1 melC2 melS1 melS2
Line
FDR= 1.01e−06
Dro (FBgn0010388)
Log2(Counts Per Million)
melC1 melC2 melS1 melS2
Line
FDR= 1.124e−06
NA (FBgn0040736)
Log2(Counts Per Million)
melC1 melC2 melS1 melS2
Line
FDR= 1.302e−06
CG18067 (FBgn0034512)
Log2(Counts Per Million)
melC1 melC2 melS1 melS2
Line
FDR= 1.482e−06

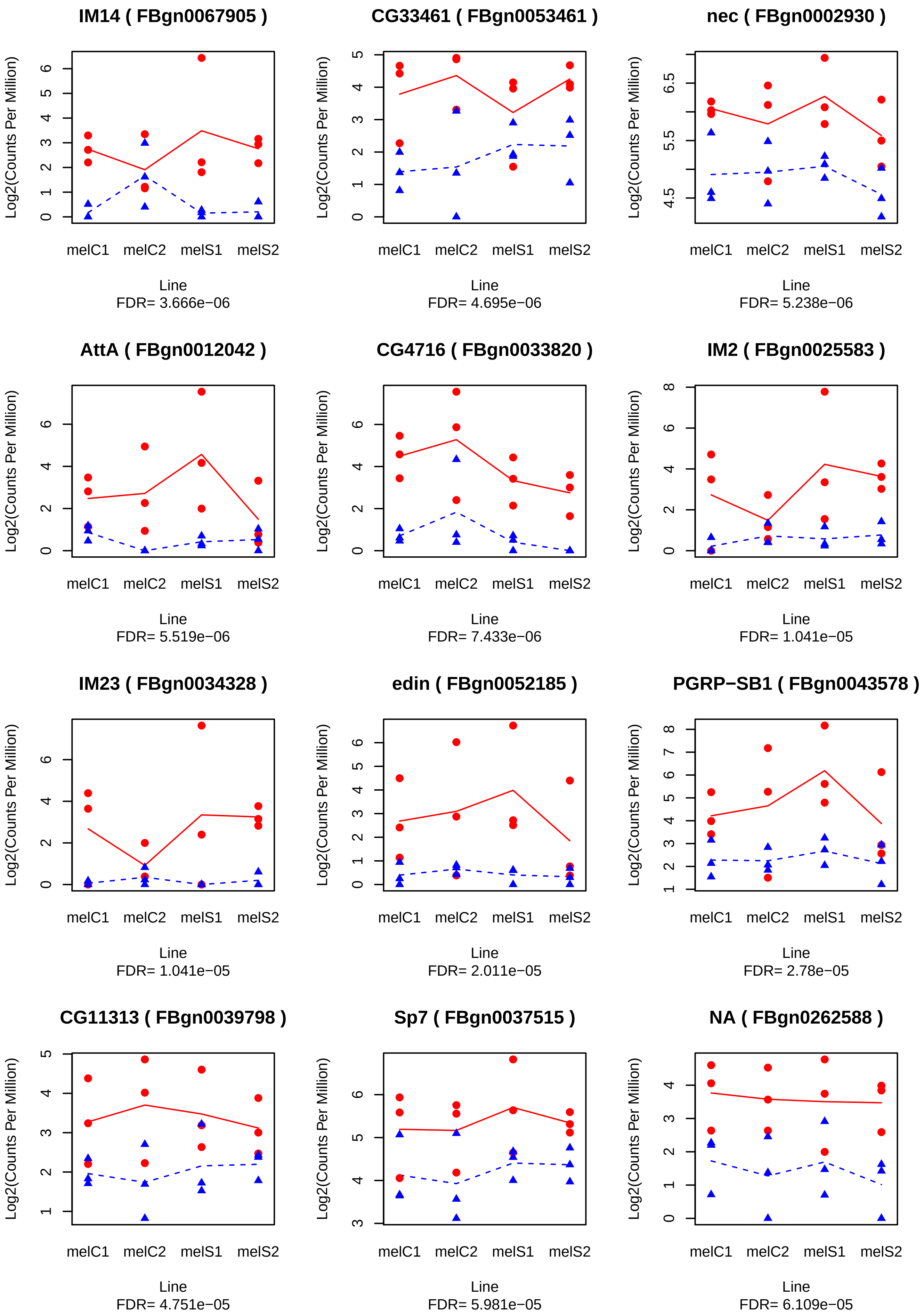
IM14 (FBgn0067905)
FDR= 3.666e−06
CG33461 (FBgn0053461)
FDR= 4.695e−06
nec (FBgn0002930)
FDR= 5.238e−06
AttA (FBgn0012042)
FDR= 5.519e−06
CG4716 (FBgn0033820)
FDR= 7.433e−06
IM2 (FBgn0025583)
FDR= 1.041e−05
IM23 (FBgn0034328)
FDR= 1.041e−05
edin (FBgn0052185)
FDR= 2.011e−05
PGRP−SB1 (FBgn0043578)
FDR= 2.78e−05
CG11313 (FBgn0039798)
FDR= 4.751e−05
Sp7 (FBgn0037515)
FDR= 5.981e−05
NA (FBgn0262588)
FDR= 6.109e−05
Log2(Counts Per Million)
Line
melC1
melC2
melS1
melS2

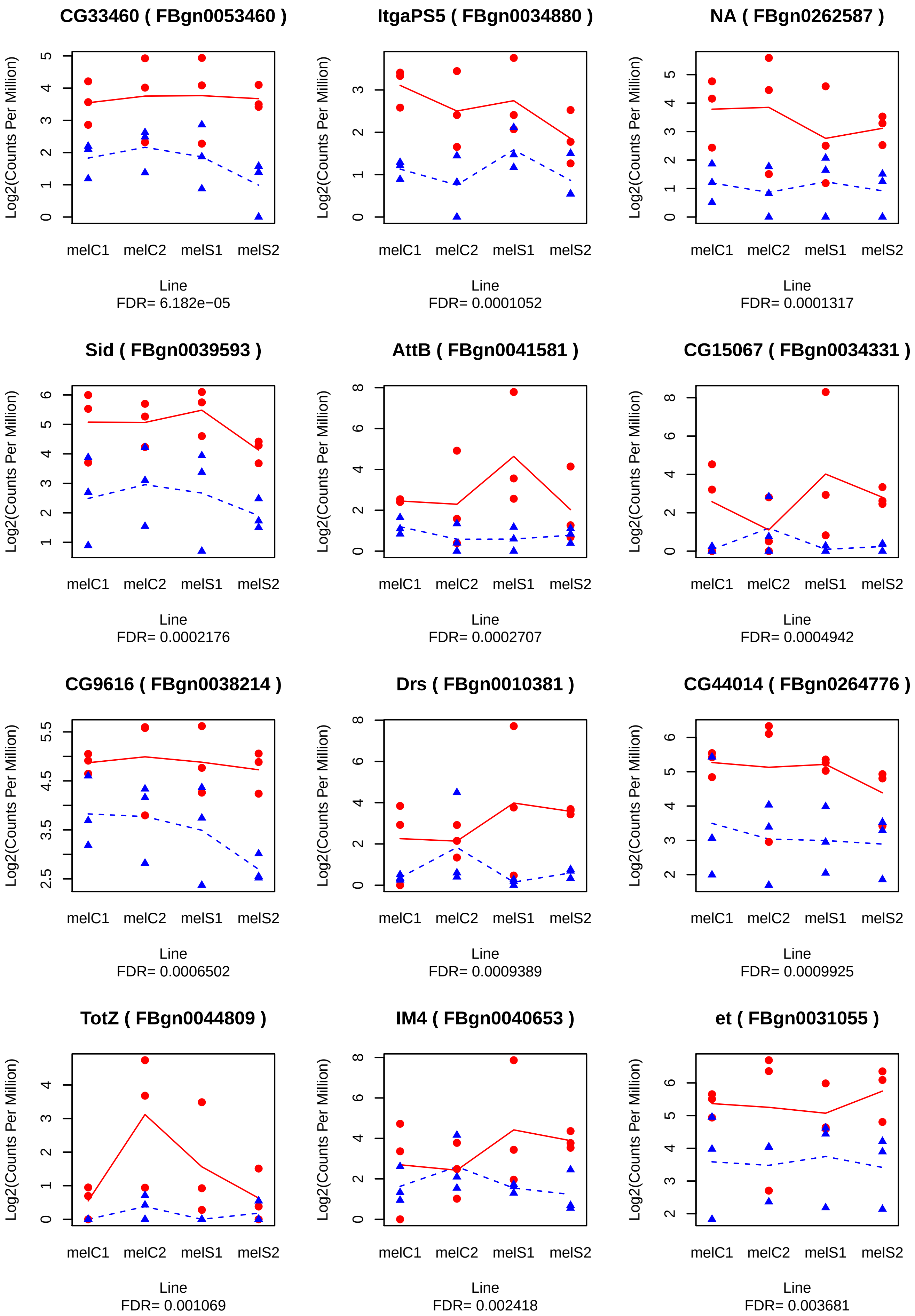
CG33460 (FBgn0053460)
Log2(Counts Per Million)
melC1 melC2 melS1 melS2
Line
FDR= 6.182e−05
ItgaPS5 (FBgn0034880)
Log2(Counts Per Million)
melC1 melC2 melS1 melS2
Line
FDR= 0.0001052
NA (FBgn0262587)
Log2(Counts Per Million)
melC1 melC2 melS1 melS2
Line
FDR= 0.0001317
Sid (FBgn0039593)
Log2(Counts Per Million)
melC1 melC2 melS1 melS2
Line
FDR= 0.0002176
AttB (FBgn0041581)
Log2(Counts Per Million)
melC1 melC2 melS1 melS2
Line
FDR= 0.0002707
CG15067 (FBgn0034331)
Log2(Counts Per Million)
melC1 melC2 melS1 melS2
Line
FDR= 0.0004942
CG9616 (FBgn0038214)
Log2(Counts Per Million)
melC1 melC2 melS1 melS2
Line
FDR= 0.0006502
Drs (FBgn0010381)
Log2(Counts Per Million)
melC1 melC2 melS1 melS2
Line
FDR= 0.0009389
CG44014 (FBgn0264776)
Log2(Counts Per Million)
melC1 melC2 melS1 melS2
Line
FDR= 0.0009925
TotZ (FBgn0044809)
Log2(Counts Per Million)
melC1 melC2 melS1 melS2
Line
FDR= 0.001069
IM4 (FBgn0040653)
Log2(Counts Per Million)
melC1 melC2 melS1 melS2
Line
FDR= 0.002418
et (FBgn0031055)
Log2(Counts Per Million)
melC1 melC2 melS1 melS2
Line
FDR= 0.003681

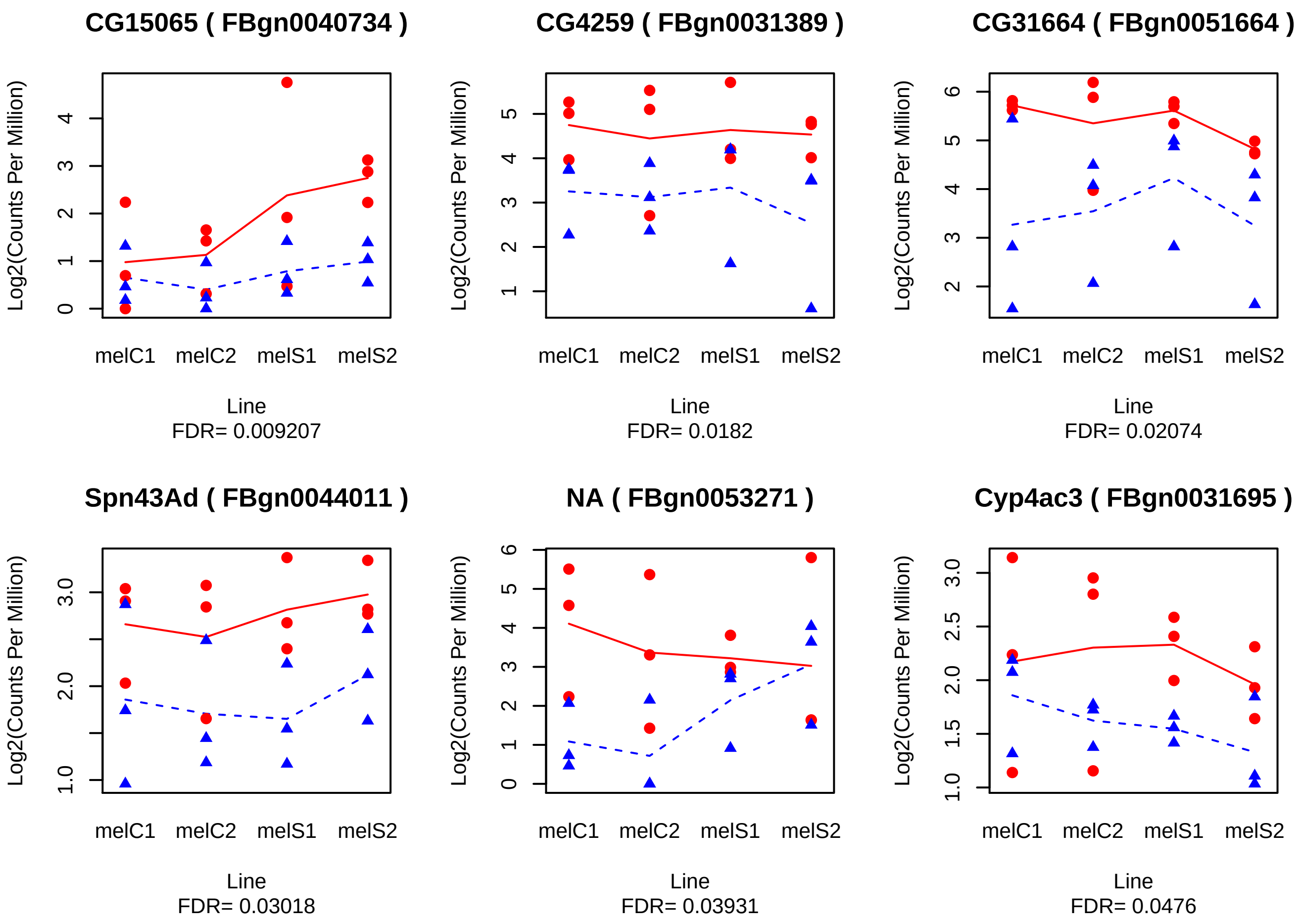
CG15065 (FBgn0040734)
CG4259 (FBgn0031389)
CG31664 (FBgn0051664)
Log2(Counts Per Million)
melC1
melC2
melS1
melS2
Line
FDR= 0.009207
FDR= 0.0182
FDR= 0.02074
Spn43Ad (FBgn0044011)
NA (FBgn0053271)
Cyp4ac3 (FBgn0031695)
FDR= 0.03018
FDR= 0.03931
FDR= 0.0476
