# Supplementary material for: Inter- and intra-species variation in genome-wide gene expression of Drosophila in response to parasitoid wasp attack
Source: BMC Genomics. 2017 Apr 27;18:331. doi: 10.1186/s12864-017-3697-3 (PMC5406980; doi:10.1186/s12864-017-3697-3)
Supplement: Supplementary file 6 — CPM Plot for D. melanogaster at 50h. Log2 counts per million of control (blue triangles) and parasitized (red circles) for D. melanogaster at 50h. (PDF 28 kb) [file 12864_2017_3697_MOESM6_ESM.pdf]

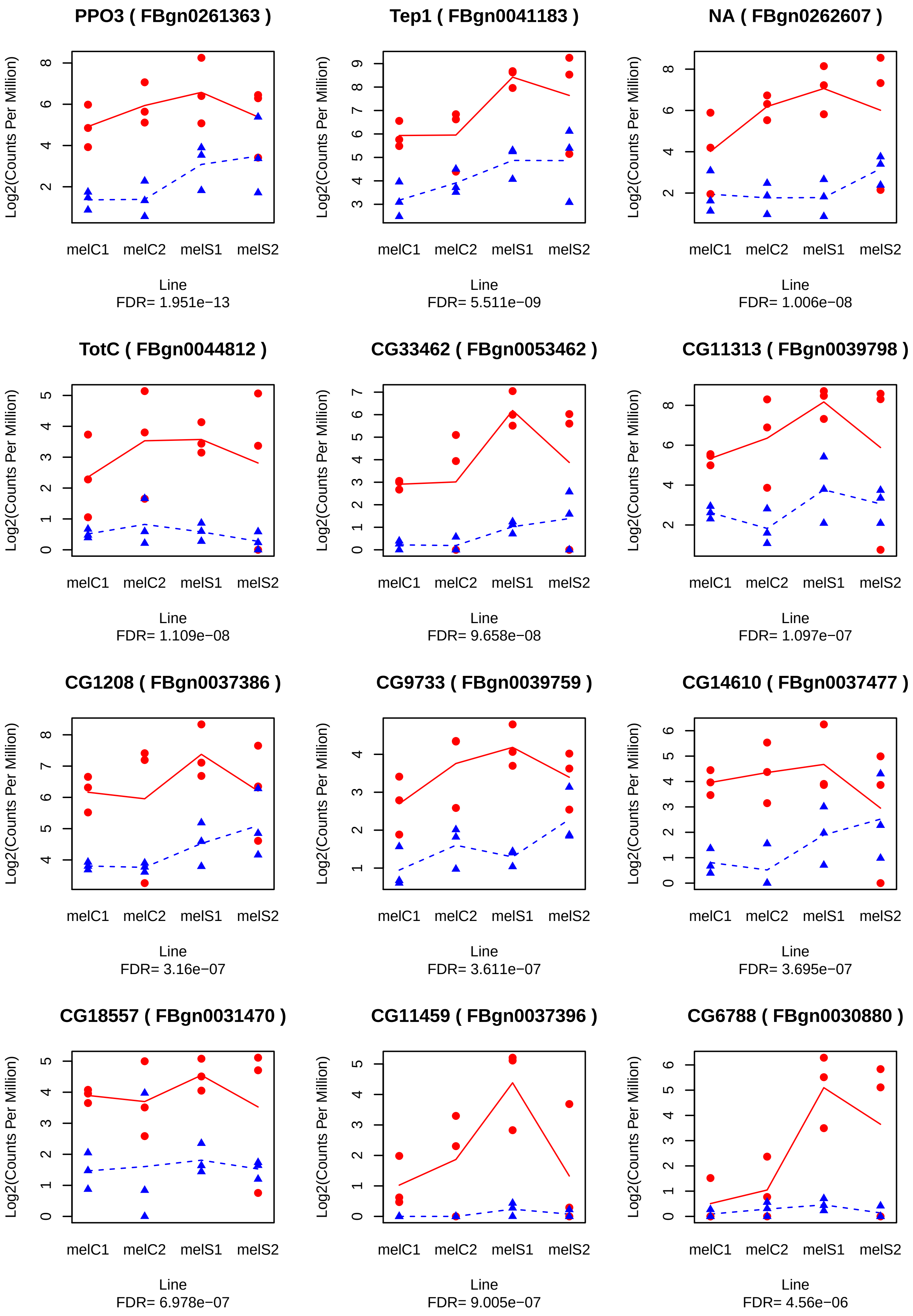
PPO3 (FBgn0261363)
Log2(Counts Per Million)
melC1 melC2 melS1 melS2
Line
FDR= 1.951e−13
Tep1 (FBgn0041183)
Log2(Counts Per Million)
melC1 melC2 melS1 melS2
Line
FDR= 5.511e−09
NA (FBgn0262607)
Log2(Counts Per Million)
melC1 melC2 melS1 melS2
Line
FDR= 1.006e−08
TotC (FBgn0044812)
Log2(Counts Per Million)
melC1 melC2 melS1 melS2
Line
FDR= 1.109e−08
CG33462 (FBgn0053462)
Log2(Counts Per Million)
melC1 melC2 melS1 melS2
Line
FDR= 9.658e−08
CG11313 (FBgn0039798)
Log2(Counts Per Million)
melC1 melC2 melS1 melS2
Line
FDR= 1.097e−07
CG1208 (FBgn0037386)
Log2(Counts Per Million)
melC1 melC2 melS1 melS2
Line
FDR= 3.16e−07
CG9733 (FBgn0039759)
Log2(Counts Per Million)
melC1 melC2 melS1 melS2
Line
FDR= 3.611e−07
CG14610 (FBgn0037477)
Log2(Counts Per Million)
melC1 melC2 melS1 melS2
Line
FDR= 3.695e−07
CG18557 (FBgn0031470)
Log2(Counts Per Million)
melC1 melC2 melS1 melS2
Line
FDR= 6.978e−07
CG11459 (FBgn0037396)
Log2(Counts Per Million)
melC1 melC2 melS1 melS2
Line
FDR= 9.005e−07
CG6788 (FBgn0030880)
Log2(Counts Per Million)
melC1 melC2 melS1 melS2
Line
FDR= 4.56e−06

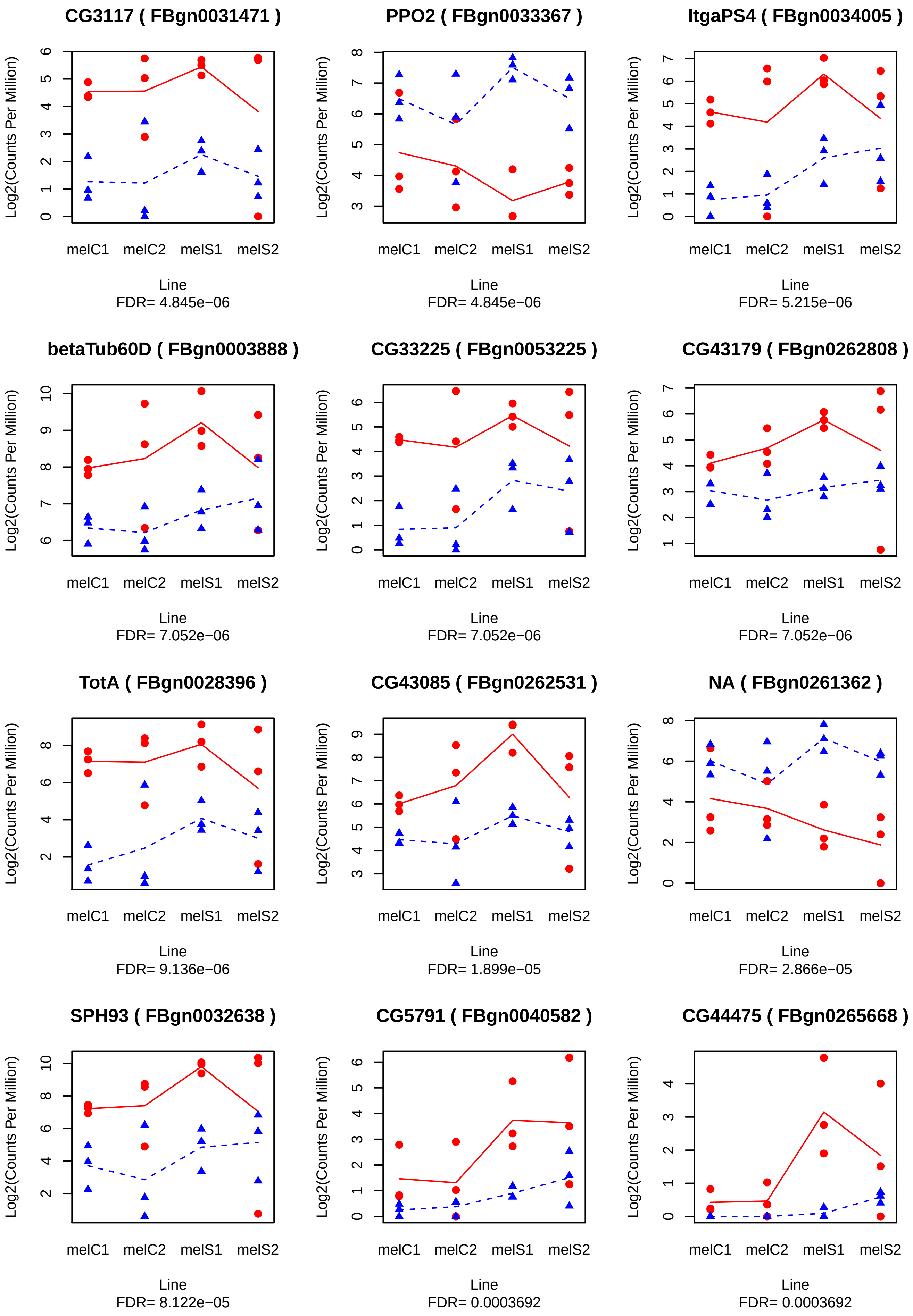
CG3117 (FBgn0031471)
Log2(Counts Per Million)
melC1 melC2 melS1 melS2
Line
FDR= 4.845e−06
PPO2 (FBgn0033367)
Log2(Counts Per Million)
melC1 melC2 melS1 melS2
Line
FDR= 4.845e−06
ItgaPS4 (FBgn0034005)
Log2(Counts Per Million)
melC1 melC2 melS1 melS2
Line
FDR= 5.215e−06
betaTub60D (FBgn0003888)
Log2(Counts Per Million)
melC1 melC2 melS1 melS2
Line
FDR= 7.052e−06
CG33225 (FBgn0053225)
Log2(Counts Per Million)
melC1 melC2 melS1 melS2
Line
FDR= 7.052e−06
CG43179 (FBgn0262808)
Log2(Counts Per Million)
melC1 melC2 melS1 melS2
Line
FDR= 7.052e−06
TotA (FBgn0028396)
Log2(Counts Per Million)
melC1 melC2 melS1 melS2
Line
FDR= 9.136e−06
CG43085 (FBgn0262531)
Log2(Counts Per Million)
melC1 melC2 melS1 melS2
Line
FDR= 1.899e−05
NA (FBgn0261362)
Log2(Counts Per Million)
melC1 melC2 melS1 melS2
Line
FDR= 2.866e−05
SPH93 (FBgn0032638)
Log2(Counts Per Million)
melC1 melC2 melS1 melS2
Line
FDR= 8.122e−05
CG5791 (FBgn0040582)
Log2(Counts Per Million)
melC1 melC2 melS1 melS2
Line
FDR= 0.0003692
CG44475 (FBgn0265668)
Log2(Counts Per Million)
melC1 melC2 melS1 melS2
Line
FDR= 0.0003692

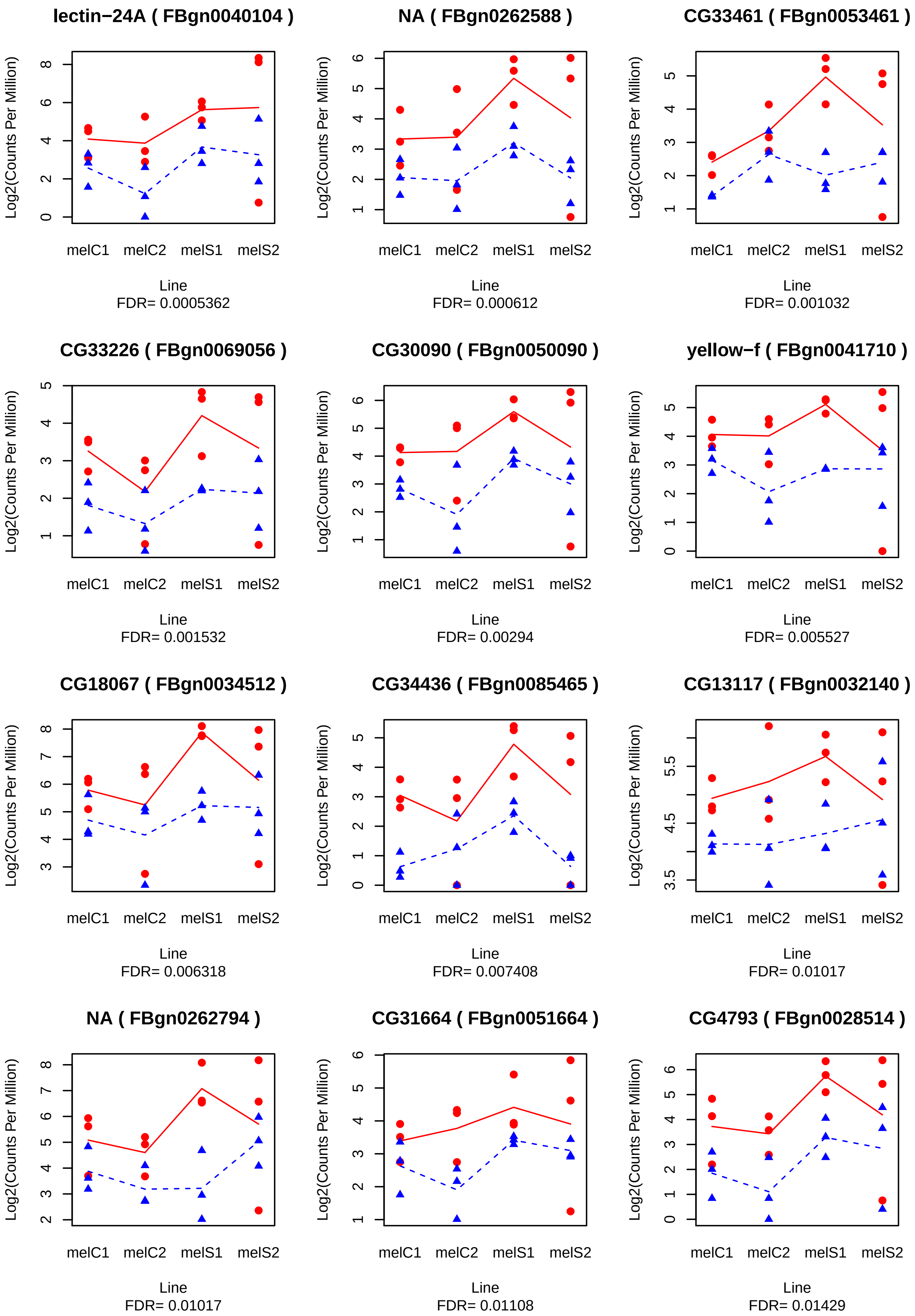
lectin−24A (FBgn0040104)
Log2(Counts Per Million)
melC1 melC2 melS1 melS2
Line
FDR= 0.0005362
NA (FBgn0262588)
Log2(Counts Per Million)
melC1 melC2 melS1 melS2
Line
FDR= 0.000612
CG33461 (FBgn0053461)
Log2(Counts Per Million)
melC1 melC2 melS1 melS2
Line
FDR= 0.001032
CG33226 (FBgn0069056)
Log2(Counts Per Million)
melC1 melC2 melS1 melS2
Line
FDR= 0.001532
CG30090 (FBgn0050090)
Log2(Counts Per Million)
melC1 melC2 melS1 melS2
Line
FDR= 0.00294
yellow−f (FBgn0041710)
Log2(Counts Per Million)
melC1 melC2 melS1 melS2
Line
FDR= 0.005527
CG18067 (FBgn0034512)
Log2(Counts Per Million)
melC1 melC2 melS1 melS2
Line
FDR= 0.006318
CG34436 (FBgn0085465)
Log2(Counts Per Million)
melC1 melC2 melS1 melS2
Line
FDR= 0.007408
CG13117 (FBgn0032140)
Log2(Counts Per Million)
melC1 melC2 melS1 melS2
Line
FDR= 0.01017
NA (FBgn0262794)
Log2(Counts Per Million)
melC1 melC2 melS1 melS2
Line
FDR= 0.01017
CG31664 (FBgn0051664)
Log2(Counts Per Million)
melC1 melC2 melS1 melS2
Line
FDR= 0.01108
CG4793 (FBgn0028514)
Log2(Counts Per Million)
melC1 melC2 melS1 melS2
Line
FDR= 0.01429

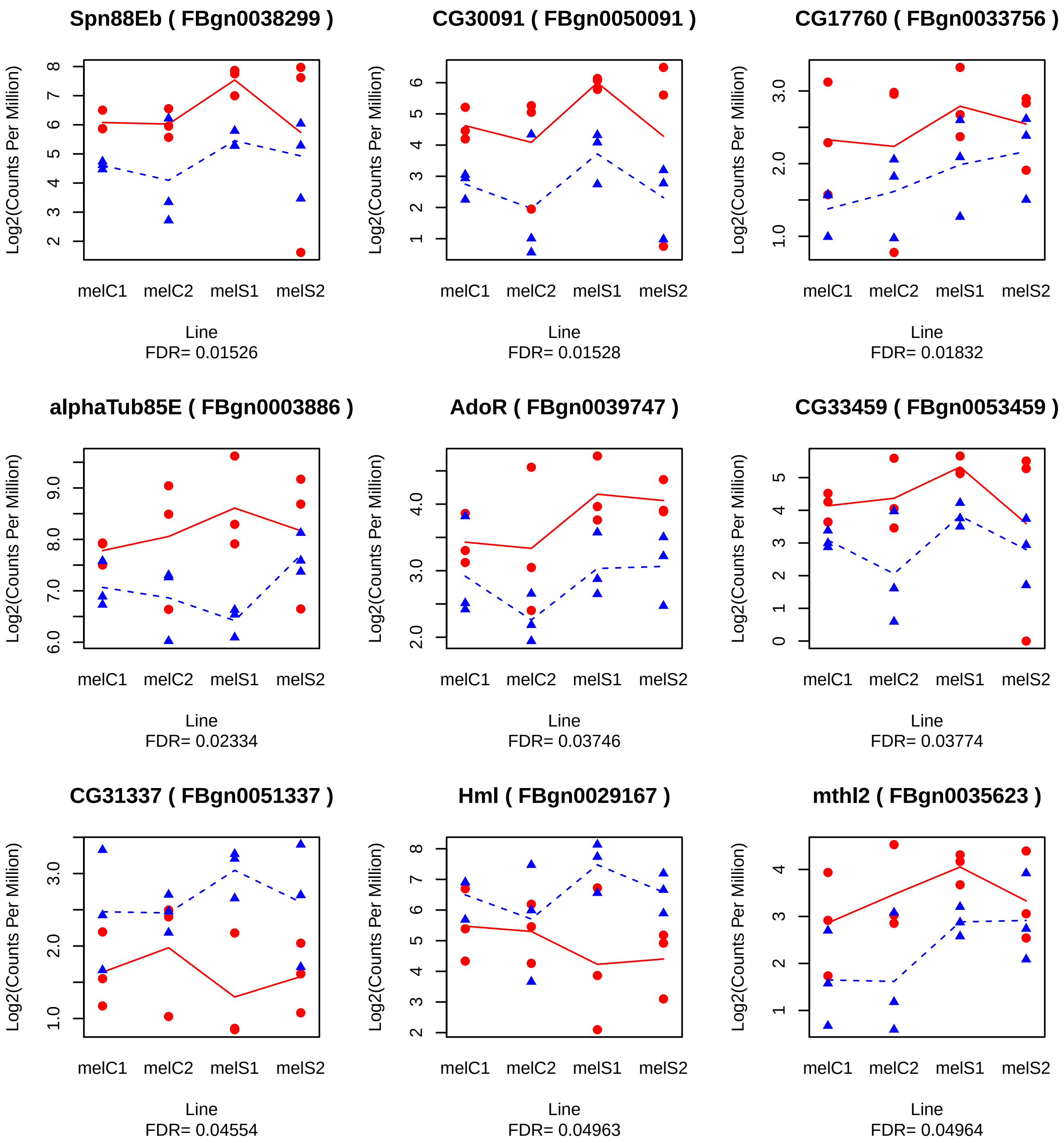

Spn88Eb (FBgn0038299)
Log2(Counts Per Million)
melC1
melC2
melS1
melS2
Line
FDR= 0.01526
CG30091 (FBgn0050091)
Log2(Counts Per Million)
melC1
melC2
melS1
melS2
Line
FDR= 0.01528
CG17760 (FBgn0033756)
Log2(Counts Per Million)
melC1
melC2
melS1
melS2
Line
FDR= 0.01832
alphaTub85E (FBgn0003886)
Log2(Counts Per Million)
melC1
melC2
melS1
melS2
Line
FDR= 0.02334
AdoR (FBgn0039747)
Log2(Counts Per Million)
melC1
melC2
melS1
melS2
Line
FDR= 0.03746
CG33459 (FBgn0053459)
Log2(Counts Per Million)
melC1
melC2
melS1
melS2
Line
FDR= 0.03774
CG31337 (FBgn0051337)
Log2(Counts Per Million)
melC1
melC2
melS1
melS2
Line
FDR= 0.04554
Hml (FBgn0029167)
Log2(Counts Per Million)
melC1
melC2
melS1
melS2
Line
FDR= 0.04963
mthl2 (FBgn0035623)
Log2(Counts Per Million)
melC1
melC2
melS1
melS2
Line
FDR= 0.04964
