# Supplementary material for: Inter- and intra-species variation in genome-wide gene expression of Drosophila in response to parasitoid wasp attack
Source: BMC Genomics. 2017 Apr 27;18:331. doi: 10.1186/s12864-017-3697-3 (PMC5406980; doi:10.1186/s12864-017-3697-3)
Supplement: Supplementary file 4 [file 12864_2017_3697_MOESM13_ESM.docx]

| | |
|---|---|
| Receptor | peptidoglycan binding<br>galactose binding<br>carbohydrate binding<br>integrin |
| | extracellular region |
